# Plasmid transmission dynamics and evolution of partner quality in a natural population of *Rhizobium leguminosarum*

**DOI:** 10.1128/mbio.02497-25

**Published:** 2025-11-10

**Authors:** David Vereau Gorbitz, Chase P. Schwarz, John G. McMullen, Mario Cerón-Romero, Rebecca T. Doyle, Jennifer A. Lau, Rachel J. Whitaker, Carin K. Vanderpool, Katy D. Heath

**Affiliations:** 1 Carl R. Woese Institute for Genomic Biology, University of Illinois Urbana-Champaign, Urbana, Illinois, USA; 2 Department of Microbiology, University of Illinois Urbana-Champaign 14589, Urbana, Illinois, USA; 3 Department of Plant Biology, University of Illinois Urbana-Champaign 14589, Urbana, Illinois, USA; 4 Department of Biology, Indiana University 1772, Bloomington, Indiana, USA; 5 Department of Biology, Case Western Reserve University 2546, Cleveland, Ohio, USA; 6 Department of Biology, McMaster University 3710, Hamilton, Canada; 7 Department of Plant and Microbial Biology, University of Minnesota Twin Cities, St. Paul, Minnesota, United States; National University of Singapore, Singapore, Singapore

**Keywords:** plasmids, multipartite genomes, natural populations, horizontal gene transfer, symbiosis, host–microbe interactions, genomics, evolution

## Abstract

Many bacterial traits important to host–microbe symbiosis are determined by genes carried on extrachromosomal replicons, such as plasmids, chromids, and integrative and conjugative elements. Multiple such replicons often coexist within a single cell and, due to horizontal mobility, have patterns of variation and evolutionary histories that are distinct from each other and from the bacterial chromosome. In nitrogen-fixing *Rhizobium*, genes carried on multiple plasmids make up a third of the genome, are necessary for the formation of symbiosis, and underlie bacterial traits, including host plant benefits. Thus, the genomics and transmission of plasmids in *Rhizobium* underlie the ecology and evolution of this important model symbiont. Here, we leverage a natural population of clover-associated *Rhizobium* in which partner quality has declined in response to long-term nitrogen fertilization. We use 62 novel, reference-quality genomes to characterize 256 replicons in the plasmidome and study their genomics and transmission patterns. We find that, of the four most frequent plasmid types, two (types II and III) have more stable size, larger core genomes, and track the chromosomal phylogeny (display more vertical transmission), while others (type I and type IV, or symbiosis plasmid, pSym) vary substantially in size and shared gene content and have phylogenies consistent with frequent horizontal transmission. We also find differentiation in pSym subtypes driven by long-term nitrogen fertilization. Our results highlight the variation in plasmid transmission dynamics within a single symbiont and implicate plasmid horizontal transmission in the rapid evolution of partner quality.

**IMPORTANCE:**

Understanding how bacterial genes move through natural populations is critical for understanding how bacterial traits evolve. Nitrogen-fixing bacteria *Rhizobium leguminosarum* live in symbiosis with plants and are a model for studying plasmid transmission and how mobile genetic elements impact the evolution of bacteria and plants. Here, we characterize the genomes of a natural bacterial population, then use novel approaches to show that mechanisms of gene transmission vary across multiple plasmid types that coexist within *R. leguminosarum* cells. We find that changes in the frequency of specific pSym types are associated with the decline of symbiotic partner quality in strains isolated from environments undergoing long-term fertilization. These results underscore the importance of plasmid transmission and evolution in shaping ecosystem processes like nitrogen cycling via bacterial-plant symbiosis. Our study provides a framework for probing plasmid dynamics within natural bacterial populations and how plasmid transmission affects genetic diversity and ecological interactions in bacteria.

## INTRODUCTION

Predictive models of bacterial trait evolution require a comprehensive understanding of how bacterial genes are inherited in natural populations. Bacterial traits arise and evolve via point mutations, gene duplication, recombination, and structural variants like transposons (1). Various mechanisms of horizontal gene transfer (HGT) lead to homologous and non-homologous recombination, which result in evolutionary histories that differ from those expected under vertical inheritance through cell division (2–5). At the core of all these processes are the vertical and horizontal transfer of genes and extrachromosomal elements, such as plasmids (6, 7), between strains. Diverse plasmids are characterized by distinct patterns of mutation, recombination, gene flow, and co-inheritance, which in turn influence how traits on these elements evolve through time (8–10). Bacterial genomes having two or more independent replicons—multipartite genomes—allow us to test how patterns of gene content, sequence similarity, size variation, and horizontal transmission vary across multiple extra-chromosomal elements within a single lineage of bacteria and thereby impact trait evolution.

Species with multipartite genomes are prevalent in nature and fill important ecological niches, including as opportunistic pathogens/mutualists and obligate endosymbionts. Multipartite genomes are particularly enriched in Pseudomonadota, but have been found in distant phyla, including Cyanothece, Leptospira, and Deinococcus (11). The plasmids of multipartite genomes carry genes in symbionts that expand their hosts’ metabolism, as well as genes that confer resistance to antibiotics and heavy metal toxicity (12–17). Plasmids can replicate independently of the chromosome, be transmitted in whole or in part between bacteria via conjugation, and increase the size and diversity of a pangenome (the repertoire of core and variable genes found in either all or some members of the species, respectively) (18–21). Moreover, this pangenomic variation is due in part to gene gain and loss via the action of mobile genetic elements within the pangenome, often found on plasmids (22). Due to HGT, plasmids can have evolutionary histories that are quite distinct from those of their bacterial chromosomes (23), effectively decoupling the fitness interests of plasmids and chromosomes and even driving coevolutionary dynamics among the elements within a single cell (24, 25). Given the importance of plasmids in symbionts and their ability to modify their hosts’ ecology, there is a critical need for a plasmid-centric approach in natural bacterial populations.

Genomic variation in bacterial pangenomes, including single-nucleotide polymorphisms (SNPs), indels, translocations, inversions, and duplications (26), is often concentrated on plasmids (27). Yet approaches to studying genetic variation using high-throughput shotgun sequencing tend to exclude or minimally investigate plasmids (28). For instance, homologous genes are used to calculate common markers of phylogenetic distance, such as average nucleotide identity (ANI) and percentage of shared SNPs—by definition ignoring vast pangenomic variation often present on plasmids. Reference-based assemblies fail to fully capture presence-absence diversity and can miss major genome rearrangements, meaning that much of the genetic variation driving genome evolution can be missed or misassembled (29, 30). Non-reference-based, *de novo* assembly of closed, circular replicons is made difficult by repetitive elements in bacterial genomes and particularly plasmids, resulting in genome fragmentation (31) and hindering the assembly and analysis of plasmids and pangenomes (32, 33). Harnessing long-read technology to assemble populations of closed, reference-quality genomes enables pangenome analysis of genetic diversity in natural populations—the diversity on which selection acts.

Rhizobial bacteria that fix nitrogen in symbiosis with leguminous plants (34), and clover-associated *Rhizobium leguminosarum* in particular, are powerful tools for understanding the contribution of plasmid diversity and transmission to symbiosis evolution in nature. The *R. leguminosarum* species complex was recently divided into at least 18 genospecies based on both chromosomal variation and plasmid content (35, 36); most recently, certain genospecies (*gsA, gsB, gsD*) have been classified as new taxa (*Rhizobium brockwellii*, *johnstonii,* and *beringeri,* respectively) (37). *Rhizobium* isolates are well-known to carry multiple plasmids, which can coexist in the same cell due to variation at the *repABC* operon (35, 38); thus, *repA* serves as a marker for incompatibility groups, and the same *repA* “Rh group” has been found across multiple genospecies (35). The canonical symbiosis genes that enable *Rhizobium* to interact with clover, vetch, and other host species are usually found on plasmids (“pSyms”), though the identity of the pSym varies among natural isolates, indicating HGT of this critical gene region across replicons (34, 35, 39, 40). Indeed, HGT of symbiosis plasmids results in evolutionary histories of rhizobial symbiosis genes and symbiosis plasmids that are distinct from those of the chromosome (8, 25, 36, 41, 42). Previous studies have shown that transposon-mediated transfer of the symbiosis island from *R. leguminosarum* into wild *Agrobacterium* and *Rhizobium* strains enabled nodulation and nitrogen fixation with a plant host (43, 44), and the transfer of *sym* genes can play a key role in *Rhizobium* speciation events (45, 46). Within *Rhizobium* species, mobilization of symbiosis plasmids is mediated by *Tra* family proteins, which enable conjugation (and thus HGT) and are regulated by quorum sensing (47, 48).

In previous work using a population of *Rhizobium* from the Long Term Ecological Research (LTER) site at Kellogg Biological Station (KBS) in Michigan, strains from old field communities exposed to long-term (multiple decades) nitrogen (N) fertilization were found to be less beneficial on average for clover (*Trifolium* spp.) host plants, compared to those from unfertilized control plots (49). Moreover, reference-based SNP analysis associated this decline in partner quality with differentiation at the canonical symbiosis gene region (50). Thus, beyond contributing to fundamental knowledge on plasmids, a fuller understanding of plasmid inheritance in these multi-partite genomes is critical for understanding symbiosis gene transmission and thus the role of HGT in the evolution of symbiotic traits. In this study, we generate 62 novel high-quality reference genomes of *Rhizobium* initially collected from the LTER KBS fields that were either fertilized with N (*n* = 28) or unfertilized (*n* = 34) to study plasmid dynamics in a natural bacterial population (42, 49, 50). We use this population to understand how sequence diversity, gene function, and size differ among plasmids within a pangenome (the plasmidome). We then use phylogenomic approaches to infer how the propensity for horizontal and vertical inheritance differs across plasmids in the pangenome. Finally, we study the pSym to examine the role of plasmid HGT in partner quality decline.

## RESULTS

### Delineating the plasmidome

Our chromosomal analysis indicated that 56 strains formed a single clade identified as *gsE,* four strains were *gsB*, and two additional strains (717_N and 773_N) were outside both taxa (Supplemental results; Fig. S1). To assess the diversity of extrachromosomal elements in natural populations of *Rhizobium*, we used long-read technology to sequence and assemble complete genomes *de novo* for all 62 nodule isolates from Weese et al. (49). Each strain’s assembled genome contained 1–6 extrachromosomal elements; plasmids accounted for ~29.19% of the genomes on average. In total, we identified 256 extrachromosomal elements in our bacterial population. Of those, 255 had at least one *repABC* operon, which has been used as a basis for plasmid categorization (35, 36, 51, 52), and 17 replicons had two distinct *repABC* operons. One 1.4 Mbp replicon in strain 773_N did not contain identifiable plasmid replication genes.

**Fig 1:**
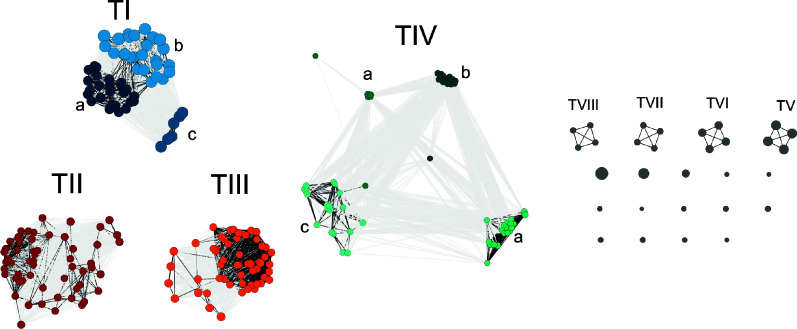
Naïve clustering reveals distinct plasmid types in the weighted, undirected network of all 256 plasmids from a population of 62 clover-associated natural isolates of *Rhizobium*. Plasmids (nodes) were grouped by Jaccard index similarity >0.1 into types. Node size indicates the size of the plasmid (bp). Darker edges indicate more pairwise similarity between nodes. Nodes were colored blue (type I), red (type II), orange (type III), and green (type IV), and shaded by subtype for types I and IV plasmids. Plasmid types with few representatives (V–VIII and singletons) were left unshaded.

**Fig 2:**
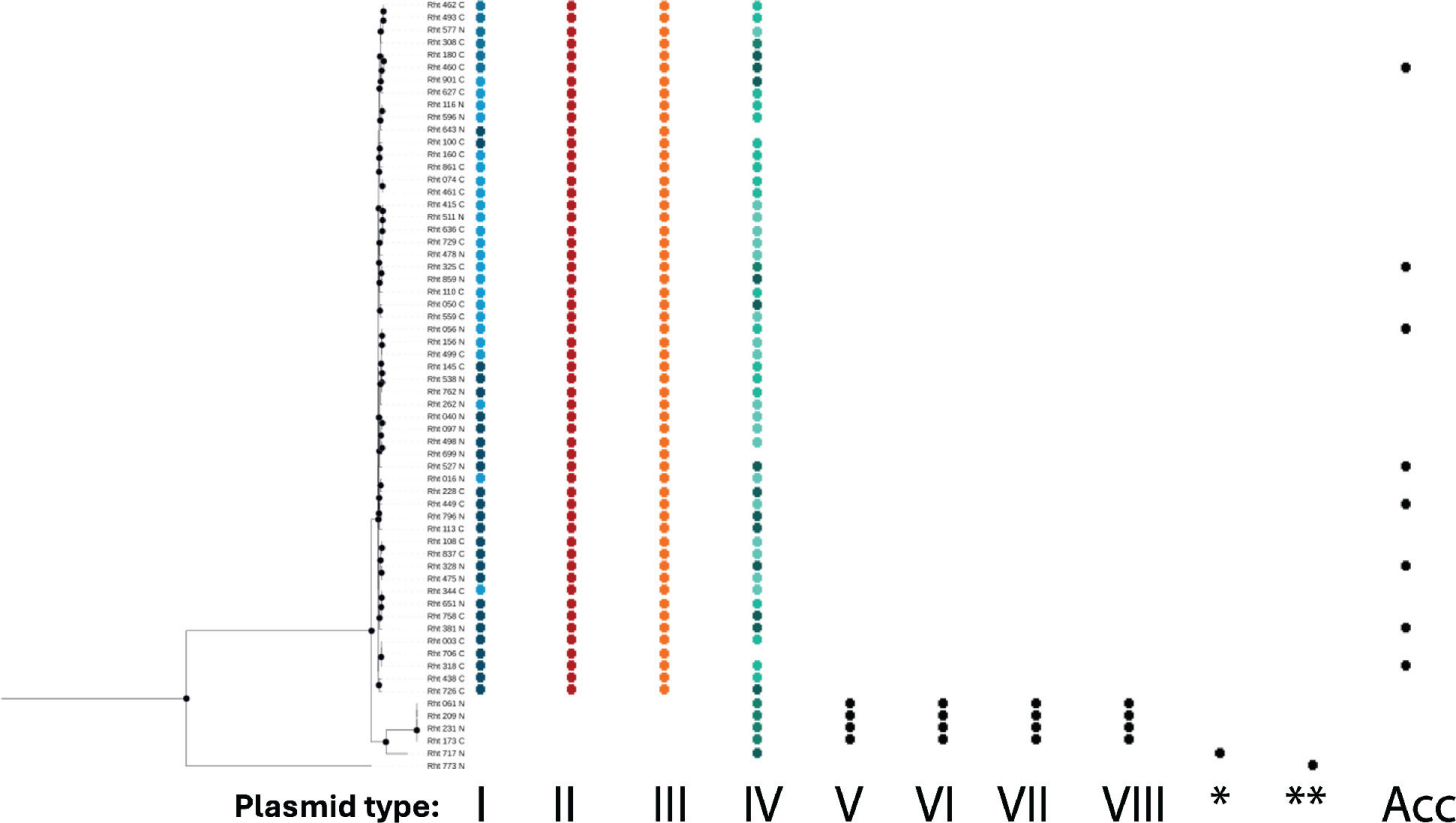
Most plasmid types are limited to only one *Rhizobium* chromosomal genospecies. Distribution of plasmid types across the concatenated core chromosomal tree from a natural population of 62 clover-associated strains. Nodes with bootstrap values over 85 are indicated by a black circle. Subtypes within types are represented by shape for type I: blue (type I-a), light blue (type I-b), and dark blue (type I-c), and type IV: light green (type IV-a), dark green (type IV-b), pastel green (type IV-c), and green (type IV-d). Six replicons from strain 717_N (*) and a single 1.4 Mbp replicon from strain 773_N (**) are each represented by a single dot. Singleton uncategorized plasmids are annotated as accessory (Acc) plasmids.

To categorize all plasmids into discrete groups, we used a k-mer approach, which considers core and non-core regions of all plasmids (as opposed to using only core gene-coding regions present in all plasmids). We calculated the k-mer signatures for all 256 replicons and generated a weighted undirected network component graph based on the Jaccard Index for all-vs-all combinations. This more inclusive, k-mer-based approach is especially important since there is little overlap in genes across all plasmids. Of 256 plasmids, most (226 or 88%) fell into one of four plasmid clusters (hereafter types I, II, III, and IV; Fig. 1), while the few remaining plasmids (16 or 6%) fell into an additional four types (hereafter types V, VI, VII, and VIII). Plasmids were numbered based on sample number, then size within the population. The distribution of these plasmid types in our study corresponds to the different genospecies in the chromosome phylogeny (Fig. 2). Types I, II, and III plasmids were present in all 56 *gsE* strains (Fig. 2), while the plasmid type groups V, VI, VII, and VIII were present in all four *gsB* strains, indicating that these plasmid types track with chromosomal genospecies. Type IV plasmids, by contrast, were found in 58/62 strains across both genospecies (i.e., *gsB* and *gsE*). Like their associated *gsB* chromosomes, types V–VIII plasmids contain little genomic diversity (Table 1).

**TABLE 1:** Summary of *Rhizobium* plasmids featured in this study^*a*^

Plasmid type	N	Present in	Size (Mbp)	Core length (Mbp)*^a^	Core genes*	Nucleotide diversity (π)*	ANI (%)*	Rh type
I	56	gsE	0.92–1.43	0.61	510	0.015	97.94	1
II	56	gsE	0.51–0.63	0.39	335	0.02	97.32	2
III	56	gsE	0.56–0.73	0.50	421	0.017	97.89	4
IV	58	gsE, gsB, Rht_717_N	0.26–0.46	0.05	49	0.021	95.25	4, 6, 7, 8
V	4	gsB	0.93	0.93	587	0	100	1
VI	4	gsB	0.67	0.67	419	0	100	2
VII	4	gsB	0.39	0.39	219	0	100	5
VIII	4	gsB	0.35	0.35	246	0	100	3
IX	1	Rht_717_N	1.10	–^*b*^	–	–	–	1
X	1	Rht_717_N	0.61	–	–	–	–	2
XI	1	Rht_717_N	0.31	–	–	–	–	3
XII	1	Rht_717_N	0.29	–	–	–	–	9
XIII	1	Rht_717_N	0.21	–	–	–	–	4

^*a*^ For plasmids with more than one representative, we report the range of plasmid size, core length, number of core genes, nucleotide diversity (π), and ANI. Rh type is based on the *repABC* sequence as defined in previous studies (35). *Statistics calculated on plasmid types with at least more than 1 sample.

^*b*^ "–", not applicable.

To identify the pSym in our population of clover-associated *Rhizobium* (Table S1), we next examined the presence of the canonical symbiosis genes (i.e., *nif*, *fix*, and *nod*) and found them limited to type IV plasmids in our annotated genomes. Interestingly, four of the 62 strains lacked the pSym (as previously found based on short-read assembled genomes), further corroborating that this non-essential element could be lost in strains present within a naturally occurring population (50). Given that all 62 strains were isolated from nodules, it is not known whether this loss occurred in culture or whether these isolates co-infected nodules with symbiotically capable strains (53, 54).

Of the remaining smaller plasmid clusters, 14 unique plasmids did not group with any other plasmid within the population (Fig. 1). Of these singleton replicons, six were found in strains outside the main *gsE/gsB* clades (717_N and 773_N); five of the singleton replicons belonged to strain 717_N (types IX, X, XI, XII, and XIII), and one belonged to strain 773_N (Fig. 2). The other eight singleton plasmids (hereafter, accessory plasmids) were in strains scattered across the *gsE* phylogeny (Fig. 2; Table S2).

### Gene content differentiates plasmid types

**Fig 3:**
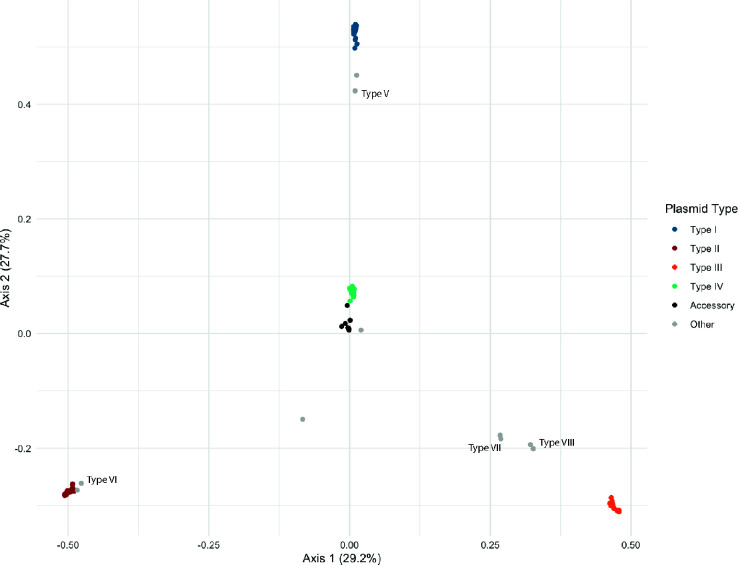
Gene content variation supports functionally distinct plasmid types. PCoA based on the presence-absence matrix of orthologous gene clusters in the *Rhizobium* plasmidome of 62 natural isolates. Each point represents a single plasmid, with points closer together indicating more shared gene content.

To visualize the difference in gene composition among plasmids, we annotated the genomes using NCBI’s Prokaryotic Genome Annotation Pipeline (PGAP) (55), assigned genes to clusters of orthologous genes (COG) groups (56), and performed a principal coordinate analysis (PCoA) with the Jaccard distance calculated from the presence-absence of orthologous gene clusters of the plasmidome. We found that all 256 plasmids formed clear clusters that correlated with plasmid types from k-mer clustering (Fig. 3), further indicating that these plasmid types differed in gene content and had distinct functional roles. In the PCoA, the pSyms (type IV) clustered together in the center between plasmids I and III, and alongside the accessory plasmids, suggesting some shared gene content among these elements. In fact, many (209) genes were shared between at least one accessory plasmid and one pSym. Further details on gene content can be found in the Supplemental results.

Although the *gsB* plasmid types V–VIII formed k-mer clusters distinct from *gsE* plasmids (Fig. 1), these four plasmids were similar to (had low Jaccard distances with), and clustered in the PCoA near (Fig. 3), *gsE* plasmid types I (V), II (VI), and III (VII and VIII) (Fig. S2A)—suggesting that these sets of elements serve analogous functions in *gsB* and *gsE*. Indeed, pairwise comparison of type I with V and type II with VI indicated 56% and 74% core gene content overlap, respectively (Fig. S2B). Interestingly, while pairwise distances between *gsE* plasmid type III and either *gsB* type VII or VIII were low (Fig. S2A), the pairwise distance between *gsB* types VII and VIII was very high (Fig. S2A); this pattern suggests that the core gene content of type III in *gsE* is comprised of a combination of the cores of types VII and VIII in *gsB*. An alignment of types VII and VIII to the type III plasmid shows large syntenic aligned blocks, consistent with shared plasmid history (Fig. S2C). While plasmid types I and V were both in Rh group 1, and II and VI were both in Rh group 2, plasmid types III, VII, and VIII belonged to three different Rh groups (4, 5, and 3, respectively) (Table 1)—indicating that plasmid gene content often, but not always, tracks the *repABC*-based Rh group.

### Mode of inheritance varies in the *Rhizobium* plasmidome

We first built phylogenies based on the (small) core set of genes shared across homologous plasmids from *gsE* and *gsB* (i.e., with *gsB* plasmids as outgroups; Fig. S3A through D). Comparing these plasmid phylogenies to the chromosome using tanglegrams and a common metric of topological discordance (Generalized Robinson-Foulds or GRF distance) (57) suggests that plasmids varied in their degree of discordance with the chromosome, with pSym being most discordant (Fig. S3A through D; higher GRF values indicate more discordance between two trees). This summary approach, however, ignores gene tree heterogeneity within the elements.

**Fig 4:**
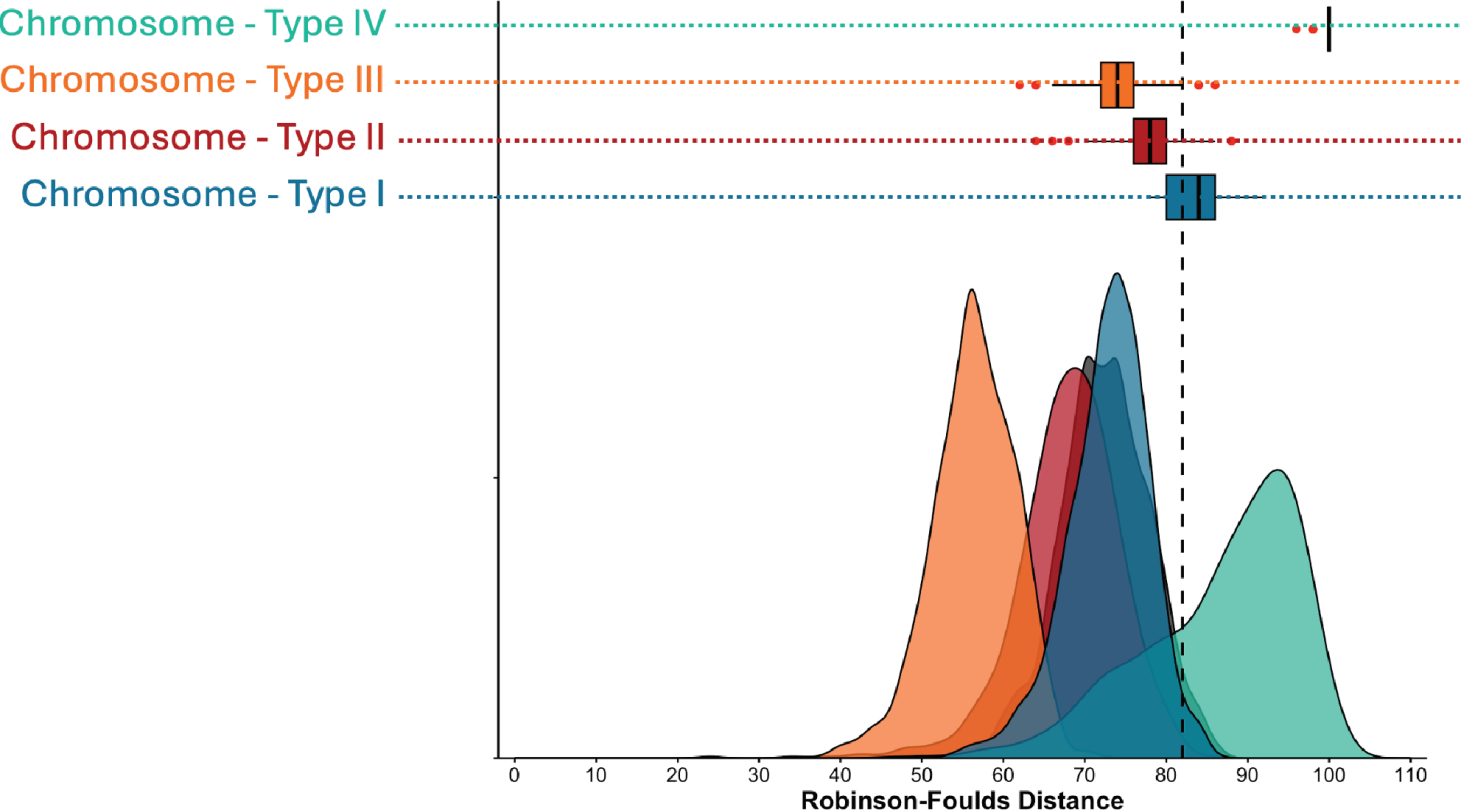
Plasmid types in *Rhizobium* have distinct levels of gene tree discordance, both within element and compared with the chromosome. Values of GRF distance closer to 0 represent lower levels of incongruence, while values closer to 100 represent higher levels of incongruence. Within-element distances for the chromosome (gray), type I plasmid (blue), type II plasmid (red), type III plasmid (orange), and type IV plasmid (green) are shown as geometric smoothed distributions along the bottom. GRF distance distributions between each plasmid and the chromosome are represented as horizontal box and whisker plots. The vertical dotted gray line represents the 95th percentile of the within-chromosome distribution.

To better account for this variability, we next focused on the set of *gsE* strains, providing a much larger core; we resampled orthologous genes, built trees from these subsamples, and generated GRF distributions for each replicon (Fig. 4). Mean GRF distance between gene trees of the chromosome was 72.46 ± standard deviation (sd) 5.40. The type I and type II plasmids had similar distributions to each other and to the chromosome (mean = 72.84 ± sd 5.08 and 68.00 ± sd 6.31, respectively)—suggesting similar levels of recombination on these three elements. By contrast, the distribution of type III plasmid GRF distances (mean = 56.41 ± sd 5.09) indicates that gene trees within this element were more concordant—suggesting more internal consistency and thus less recombination compared to the chromosome. Finally, the GRF distribution of the pSym was wider and centered at mean = 86.79 ± sd 9.46, indicating higher divergence of gene trees among the loci on this element, compared to the other four—consistent with abundant recombination of genes on the pSym.

Next, to assess the degree to which plasmids are co-transmitted with the chromosome, we computed the distributions of GRF distances when gene trees from each plasmid were compared to those from the chromosome. The distributions of GRF values for plasmid types II and III with the chromosome (horizontal box plots) fell below the 95th percentile of the chromosome-chromosome GRF distance distribution (vertical line, Fig. 4), suggesting that the discordance between genes on plasmid II/III and the chromosome is similar to that between two random sets of chromosomal genes (also see Fig. S3B and C). By contrast, the chromosome-type I gene distribution (mean = 83.24 ± sd 2.97) fell above the 95th percentile of the chromosomal gene distribution (Fig. 4), suggesting decoupled evolutionary histories of the type I plasmid and the chromosome (Fig. S3A). Finally, GRF distances between genes on the chromosome and the pSym were particularly high (mean = 99.56 ± sd = 0.83, with 881/1,000 resamplings resulting in a maximum GRF = 100; Fig. 4), also suggesting high levels of horizontal transmission of the pSym core genes relative to the chromosome.

**Fig 5:**
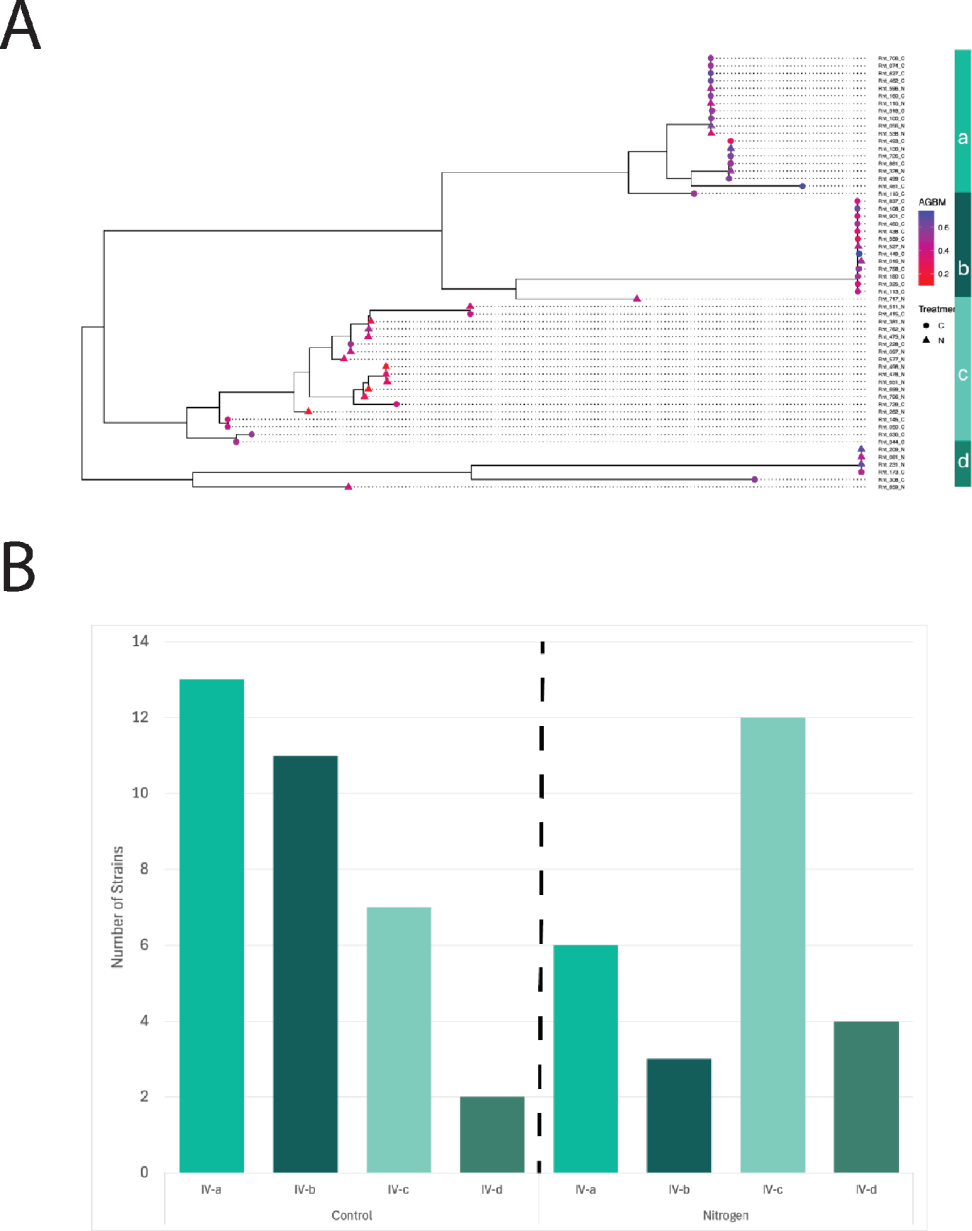
Distinct pSym subtypes differ in symbiotic partner quality and differentiate nitrogen-fertilized and control environments. (**A**) Inferred phylogenetic tree of the type IV plasmid (pSym) based on concatenated core gene alignment, with nodes labeled with aboveground biomass (AGBM as color gradient) and plot treatment of origin (C for control or N for N-fertilized). Type IV subtypes (a–d), as in Fig. 1, are indicated in shades of green on the right. (**B**) Number of strains from each type IV sub-clade isolated from either control (left) or N-fertilized (right) plots from the KBS LTER.

Indeed, we found clear cases of movement of the pSym subtypes across distinct chromosomal lineages in our population. First, although strain 717_N is more closely related to *gsB* than *gsE* at the chromosome (Fig. S1) and shares no other plasmids with either group (Fig. 2), the 717_N pSym falls alongside a group of high-quality *gsE* strains in the pSym tree (clade IV-b, Fig. 5A). Similarly, although the *gsB* and *gsE* chromosomal clades generally have distinct plasmidomes, including the pSyms (Fig. 1 and 2), two *gsE* strains (308_C and 859_N, Fig. S1) were found to carry *gsB*-like pSyms (clade IV-d; Fig. 5A) indicating cross-genospecies HGT.

### Variable-size distributions in *Rhizobium* plasmids

**Fig 6:**
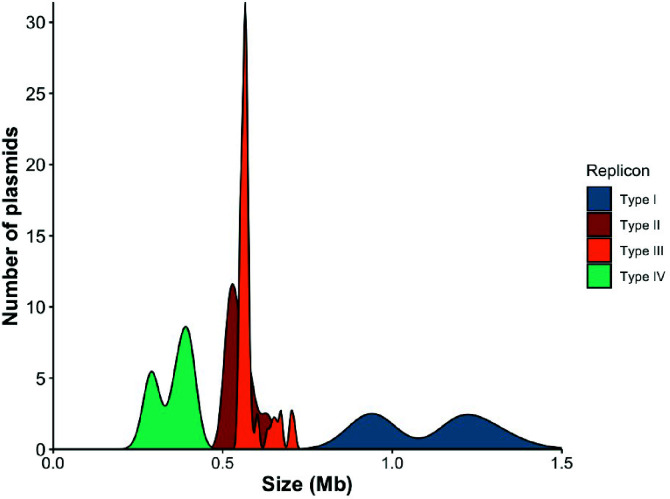
The four major plasmid types (I–IV) in *Rhizobium* genospecies (*gsE*) have distinct size distributions. Distributions of types I–III include plasmids from all 56 *gsE* strains, whereas the type IV distribution includes 58 type IV plasmids from *gsE* and *gsB* strains, as well as 717_N.

Within our best-represented genospecies (*gsE*), plasmid types varied in size and in the shape of their size distribution (Fig. 6). Type I plasmids were the largest in our population, had the largest size variation, and had a bimodal distribution centered at either ~0.9 Mbp or ~1.2 Mbp. Type II and type III plasmids were similar in size, with intermediate lengths ranging from ~0.5 to 0.6 Mbp. Of the *gsE* plasmidome (types I–IV), pSyms were the smallest (~0.2–0.4 Mbp). Notably, this symbiosis gene location is distinct from the reference genome WSM1325 (*gsH*), in which the symbiosis genes are found on the largest plasmid (34).

Comparing the size variation within the type I plasmid (Fig. 6) with its core length, content, and phylogeny (Table 1) allows us to reconstruct the history of how dramatic changes in plasmid size have evolved in this element. The three type I k-mer subtypes (I-a, I-b, I-c; Fig. 1 and 2) map onto three distinct groups in the phylogeny built on the full type I core (Fig. S4A). Both type I-b and I-c plasmids are 1.2 Mbp in length, while type I-a plasmids are 0.9 Mbp long; however, types I-b and I-c contain distinct gene content (Fig. S4B). All of the smaller type I plasmids (subtype I-a) form a clade that diverged from I-b after a single loss of the ~0.3 Mb insertion (Fig. S4B). Thus, the independent gain and loss of large insertions is responsible for the bimodal size distribution; interestingly, these large insertions occur in similar locations along the plasmid (Fig. S4B), suggesting that type I plasmids might have specific hotspots where insertions are more likely.

### pSym (type IV) plasmid movement drives differences in partner quality

Given the evidence for abundant HGT of the pSym, and previous analysis of symbiosis-related loci (50), we next studied patterns of differentiation in the pSym (Fig. 5A). A sliding window analysis of the concatenated pSym core indicates very high levels of differentiation (F_ST_ > 0.5 [58]) between pSym subtypes a–c, with the exception of conserved, but non-syntenic, transposons and mobile phage proteins (Fig. S5A).

The pSym is characterized by a particularly small core (52.1 kb, or 49 genes; Table 1; Table S1), with which the phylogenies were inferred. Nevertheless, the phylogenetic groupings (subtypes) mirror the four major k-mer clusters of type IV plasmids based on all gene content (Fig. 1), as well as PCoA of pSym gene content (Fig. S5B)—indicating that these groups do not reflect the symbiosis genes alone, but the entire plasmid diversity, including unique gene content (Fig. S5C). Nevertheless, we find abundant gene-sharing across pSym subtypes: much of the modular gene content is shared across subtypes, and the core gene set of any two subtypes (regardless of phylogenetic distance) is noticeably larger than the universal pSym core of 49 genes (Fig. S5D). For example, despite being more phylogenetically distinct and most often found in the *gsB* minor clade, nearly all IV-d genes are shared with one or more type IV sub-clades (Fig. S5D). Finally, we find similarly high GRF distributions when they are calculated using the larger core *within* pSym subtypes (Fig. S5E through H; except low diversity IV-d from *gsB*)—indicating abundant recombination among closely related pSyms.

We found striking correspondence between these pSym subtypes and the benefits of symbiosis for plant hosts, as measured in a previous common garden experiment (49). First, we used Pagel’s lambda (59) to quantify the strength of the relationship between the phylogeny of each replicon and variation in quantitative partner quality traits (aboveground biomass and chlorophyll content). We found that, unlike the chromosome and the other plasmids, the core pSym tree strongly predicted partner quality (Table S2). The pSym clades IV-a and IV-b contain mostly higher-quality strains that originated from unfertilized control plots (Fig. 5A) and feature less nucleotide diversity (π = 0.0034 and 0.0028, respectively), compared to clade IV-c, which contains an abundance of more diverse (π = 0.0053), lower-quality strains isolated from N-fertilized plots (Fig. 5A). A Chi-squared test supported a significant difference in frequency of type IV sub-clades between control and N treatments (*P* = 0.04234; Fig. 5B). By contrast to the pSym, the frequency of type I sub-clades did not differ between control (C) and N strains (*P* = 0.4153).

## DISCUSSION

Plasmid inheritance is a key process underlying bacterial trait evolution in natural and managed ecosystems. Studies of plasmid variation and transmission within local-scale, recombining populations are needed to quantify patterns of gene co-inheritance, as well as their influence on bacterial phenotypes. Here, we delineate the major types, size, gene content variation, and transmission patterns of coexisting plasmids from a single population of clover-associated *Rhizobium*. We find that *Rhizobium* plasmids vary considerably in their size, structure, and modes of transmission; some plasmids (type II, type III) appear to be primarily vertically transmitted, while others (type I, pSym) are more likely to be horizontally transmitted. Concomitant with these findings from within our best-sampled genospecies (*gsE*), we find that most of the plasmidome is delimited by chromosomal lineages. Nevertheless, the extent of this limitation varies across plasmids; for example, we find clear examples of cross-genospecies HGT of the pSym. Finally, our analysis of pSym subtypes indicates a role for pSym HGT in the decline of clover-associated partner quality in N-fertilized environments. Below, we discuss the important lessons learned from each of the major elements in our *Rhizobium* plasmidome, then finish with a holistic discussion of the potential importance of transmission variation to the evolution of bacterial traits.

### Type I plasmids

The symbiosis genes in other *Rhizobium* populations had been previously mapped to plasmids with multiple *rep* types, including Rh04, Rh06, Rh07, and Rh08 (42, 50). Indeed, in previous work (50) using reference-based assembly to the reference strain WSM1325, we assumed that the type I plasmid was the symbiosis plasmid (see discussion of the pSym below). This highlights the value of using non-reference-based genome assembly facilitated by long read sequencing, as well as naïve k-mer based clustering, for studying plasmid variation, plasmid transmission, and the evolution of plasmid-borne traits. Plasmid size variation is usually caused by differing patterns of presence and absence of genes, which is caused by homologous recombination or HGT (60, 61). Studies of plasmid evolution usually limit analyses to a handful of genes involved in plasmid replication, maintenance, and transfer (62, 63). The ability to interrogate fully closed plasmid genomes allowed the separation of core and variable content within this single plasmid type, revealing the gain and loss of large insertions that dramatically alter plasmid size and track the core phylogeny. The internal consistency of the type I plasmid (based on the comparison of core and variable content, and similar *within*-plasmid gene tree distances to those of the chromosome) at first appears at odds with evidence for recombination *between* type I plasmids and the chromosome. These results might hint at distinct mechanisms governing within-element stability versus whole-element transfer in this plasmid (e.g., homologous recombination versus conjugation), though more functional studies are required to make any generalizations.

### Type II and III plasmids

We found that genes on the type II and type III plasmids are more frequently co-inherited alongside chromosomal genes, have relatively high internal consistency of gene trees, and have a large fraction of core genes compared to the other plasmids (Table 1). This suggests that the mechanisms by which types II and III are being inherited and recombining are different compared to the types I and IV plasmids. In fact, the type III plasmid shows particularly low *within*-plasmid GRF distances, suggesting particularly low rates of recombination, though the potential mechanisms remain unclear.

 Our observation that the *gsE* type III plasmid contains the gene content of *two* other plasmids from the *gsB* group (types VII and VIII) might suggest a major evolutionary event in the history of these *Rhizobium* plasmids—either a subdivision of one (fission) or a merging of two (fusion)—and denotes a key change in how *gsE* and *gsB* strains subdivide their respective genomes. Importantly, however, the type III does not share its Rh incompatibility group (35, 51) with either of these plasmids, making historical reconstruction difficult. Chromosomal fusion is known in closely related *Agrobacterium tumefaciens* (64). The “schism hypothesis” of chromosome fission has been developed as an explanation for the evolution of multi-partite genomes (61); such processes might generate plasmid diversity as well. Fusion and fission of eukaryotic chromosomes is well-known to drive reproductive isolation and thus speciation in plants and animals (65). Understanding the impact of these processes in the diverse plasmids of *Rhizobium* and other species is paramount to further understanding bacterial genome evolution and the diversification of bacterial species.

### Type IV plasmids

The pSym is typically defined as the replicon that harbors genes necessary for symbiosis with a host plant (25). In our population, the pSym is the type IV plasmid, the smallest of the main plasmids, and the only one present in both *gsE* and *gsB* chromosomal genospecies. It is also the most variable, having a very small pool of core genes and deeply diverged lineages. Nevertheless, we treat the pSyms as one “type” of plasmid for multiple reasons. First is their clustering based on the k-mer approach we used to categorize plasmids. Though previous work has used *repABC* Rh types to categorize *Rhizobium* plasmids (35), we found that 17 pSyms in our population contain two distinct, full *repABC* operons (Fig. S5I), suggesting the need for an additional approach that reflects gene content similarity. Because plasmids with the same *repABC* groups generally cannot be maintained in the same cell (66), it is unclear what the evolutionary advantage of having two distinct copies of the operon in a single plasmid might be. Second, while some pSym lineages possessed subtype-specific gene clusters, there was abundant shared gene content in pairwise comparisons of the pSym subtypes regardless of relatedness at the core—suggesting mobility of pSym genes across the pSym phylogeny. Finally, examining all pSyms together based on gene content and function allowed us to relate pSym subtype to symbiotic partner quality and detect shifts in subtype frequency between N-fertilized and control plots (see below).

The symbiosis genes in the *R. leguminosarum* species complex are well-known to exhibit high levels of variable gene content and horizontal mobility, including being able to move through HGT or integrative and conjugative elements (25, 67, 68). Nevertheless, we found that all symbiosis genes were located in the type IV plasmids and contained *nod, nif, fix,* and *fdx* operons. Within our population, three gsE isolates lack the type IV plasmid but do have types I, II, and III plasmids. A previous study (49) had reported these three strains as poor-quality partners, and a lack of symbiosis genes explains this phenotype; how they were isolated from nodules despite lacking the pSym remains unknown. The distribution of these strains across the *gsE* phylogeny suggests that, in addition to obtaining new plasmids via HGT, these rhizobia may readily lose or shed the symbiosis plasmid. The pSym subtype type IV-d was mostly associated with a distinct chromosomal genospecies (*gsB*), and the few notable exceptions allow us to pinpoint clear cases of pSym HGT across the genospecies boundary. Previous approaches designed to detect introgression events across genospecies support shared alleles at symbiosis genes *nifB*, *nodC*, and *fixT* across *gsE* and *gsB* (35); our results suggest that the symbiosis plasmids move across these genospecies boundaries as well. Although a much more thorough functional analysis would be required to pinpoint the underlying drivers of transmission in our plasmids, extra recombination genes (L) in the pSym might explain higher levels of recombination and gene content variation in this plasmid. Nevertheless, it is interesting to consider the genetic drivers and selective forces that might reinforce, versus break up, this type of structure in chromosome-plasmid relationships through time and across environmental conditions (61, 69). Taken altogether, our results suggest that the pSym subtypes represent at least three distinct backbones of pSym types, which nevertheless are recombining with one another in nature.

Previously, differentiation of the symbiosis gene region was reported between high-quality partners in the control plots and low-quality partners in the N-fertilized plots in this *Rhizobium* population, relative to the rest of the genome (50). Here, we provide evidence that it is the entire pSym, and not just the symbiosis gene region, that is differentiated. Together with evidence for HGT of the pSym—both between chromosomal genospecies and within a single, well-sampled genospecies (*gsE*)—we infer a shift in pSym subtype frequencies with a change in the environment, rather than a gene-specific sweep at the symbiosis gene region. The shorter branch lengths, decreased recombination, and small size of the higher partner quality pSym subtypes IV-a might indicate purifying selection in control plots, compared with relaxed selection for partner quality in N-fertilized plots, as previously hypothesized (49). Our new, plasmid-centric interpretation of the genetic underpinnings of partner quality decline stems from both data type and analytical methods; our long-read-enabled full-genome assemblies reveal diversity in symbiosis gene location, duplicate pSym *repABC* types, and pSym gene content that was not previously visible. What’s more, these fully resolved plasmidome sequences, combined with novel phylogenomic analyses, allow us to quantify patterns of gene tree heterogeneity not only at the pSym but across all plasmid types and thus make inferences about the variation in transmission modes among elements within a single genome.

### Transmission in the *Rhizobium* plasmidome

The degree to which plasmid vertical versus horizontal transmission modes are governed by chromosomal mechanisms, plasmid-specific mechanisms, and/or the interaction remains to be determined (52, 70). It has long been recognized that plasmid transmission mode can evolve as the costs and benefits of conjugation-growth tradeoffs shift (71, 72), though the selective drivers in nature are not well-known. Most plasmid transmission studies take place in the lab under strongly selective conditions in one or a few laboratory or clinical strains (73, 74), whereas population genomic studies on natural diversity have historically not focused on extra-chromosomal elements, given sequencing limitations (75, 76). Studies of plasmid transmission in natural populations are rare, though others have compared gene content and plasmid diversity among non-coexisting plasmids within a single species (71, 77). Here, we establish a novel framework for studying plasmid transmission in nature and find distinct patterns of inheritance among the multiple plasmids that coexist within *Rhizobium* host cells. Some plasmids appear to move more vertically alongside the chromosome (e.g., types II and III), while others frequently move horizontally (e.g., types I and IV). Given that these plasmids co-occur in the same cells, plasmid-specific mechanisms must explain this variation, at least in part. Our findings support evolutionary conceptions of plasmids as having their own agency and fitness interests (72, 78, 79).

Rhizobia also provide a rare opportunity to study how plasmids and HGT facilitate quantitative trait evolution in natural bacterial populations. Rhizobial symbiosis genes are known to be both horizontally transmitted and selected for in nature (35, 80). In our *Rhizobium* population, we find frequent HGT of the pSym relative to the other plasmids, paired with differentiation of the pSym between N environments, suggesting environmentally dependent selection on this plasmid. The symbiosis genes in *Rhizobium* can move between plasmids (34–36, 39), and as we show here, those plasmids can vary in rates of HGT—potentially suggesting that the traits governed by these mobile loci will evolve more or less rapidly depending on their genomic location. Rapid evolution of symbiotic traits via gene-specific sweeps, whole plasmid sweeps, and even plasmid loss (81) might be advantageous in context-dependent mutualisms where the costs and benefits of symbiosis shift with the biotic and abiotic context in which partners interact (82, 83).

By integrating genomics, plasmid biology, phylogenetics, and plant–microbe interactions in wild bacteria, our study provides a framework for quantifying the relative rates of vertical and horizontal transmission among the plasmids that coexist within a single species and elucidates the role of plasmid HGT in an ecologically important symbiosis that plays a critical role in global nitrogen cycling.

## MATERIALS AND METHODS

### Strain isolation and growth

Here, we generate novel genomes for a population of 62 *Rhizobium* strains originally isolated from old field successional plots at the KBS LTER. Full methods detailing the long-term N fertilization experiment, strain isolations, and phenotypic experiments to characterize partner quality symbiosis with three clover host species are described elsewhere (49). Briefly, rhizobium strains were isolated from both N-fertilized and control plots. Fertilized plots had been supplemented with 12.3 g N m^−2^ per year granular ammonium nitrate for 22 years prior to sampling, whereas control plots remained unfertilized.

### DNA and sequencing

We grew the strain isolates in solid tryptone yeast (TY) media (5 g L^−1^ tryptone, 3 g L^−1^ yeast extract, 6 mM CaCl_2_, and 16 g L^−1^ agar) plates at 30°C for 2 days. After growth on solid media, single colonies were selected to inoculate 5 mL liquid TY media for 1 day at 30°C in a roller drum. The PacBio Nanobind CBB kit (Pacbio, San Diego, CA) was used to extract high molecular weight (50–300+ kbp) DNA from 1 mL of bacterial culture for all strain isolates. The DNA was sent for PacBio hifi long-read sequencing (84) at the W. M. Keck Center at the University of Illinois, where gDNAs were sheared with a Megaruptor 3 to an average fragment length of 10 kb, then converted to barcoded libraries with the SMRTBell Express Template Prep kit 3.0 and pooled in equimolar concentration. The pooled libraries were sequenced on 2 SMRTcell 8M on a PacBio Sequel IIe using the circular consensus sequencing (CCS) mode and a 30 h movie time. CCS analysis was done using SMRTLink V11.0 using the following parameters: ccs --min-passes 3 --min-rq 0.99 lima --hifi-preset SYMMETRIC --split-bam-named --peek-guess.

### Genome assembly and annotation

All genomes were assembled using recommended workflows in Trycycler v0.5.5 (85). Briefly, raw reads were filtered for quality using Filtlong v0.2.1, assessing both length and quality of the reads. The raw reads were then divided into 12 maximally independent subsets using the subsample function in Trycycler. Next, Trycycler uses three assembly methods (Flye v2.9.4 [86], Hifiasm v0.25 [87], and Raven v1.8.3 [88]) to generate independent whole-genome assemblies for four read subsets each. Finally, we used Trycycler to generate a consensus genome based on these 12 assemblies, followed by manual curation to ensure consistent genome structure across assemblies. Genomes were all closed, and all individual contigs on each assembly represent a closed replicon. Genomes were then annotated using NCBI’s PGAP v6.3 (55).

### Classification of plasmids

We split replicons out of each assembled genome (see Results) by separating individual contigs into individual fasta files. Then we used those individual contigs as input data for sourmash v4.8.15 (89) to generate and compare k-mer signatures and calculate pairwise Jaccard Index (JI) values using the parameters: sketch dna -p scaled=10000, k=31, compare -p 8. Signature tables were then imported into Cytoscape, and component graphs were created using a minimum JI value of 0.1 to delineate clusters; this cutoff was chosen based on similar analyses of *Rhizobium*, *Agrobacterium*, *Bradyrhizobium*, as well as a global plasmid analysis (9, 38, 90). We classified the resulting clusters as plasmid “types,” then layered this plasmid presence onto phylogenetic trees using “ggtree” and “ggplot” libraries in R (version 4.3.2 “eye holes”). We used the package Popgenome v2.7.2 to calculate nucleotide diversity and ANI, and fixation index for aligned core regions of each plasmid type.

### Phylogenetic trees

We used a custom SPINE-Nucmer pipeline (https://github.com/Alan-Collins/Spine-Nucmer-SNPs) to generate core genome alignments for all 62 genomes (core genes were all chromosomal), all chromosomes (resulting in the same core), and subsequently for each plasmid type. For each, core components were concatenated, aligned using Mummer v3.2, and used to generate phylogenetic trees in IQTree2 v2.4 with parameters: -bb 10000 -st DNA. Tanglegrams were generated by loading phylogenetic trees in contree format into RStudio and using the function “cophylo” to plot the tanglegrams. GRF distances for tanglegrams were calculated using the “treedist” function.

### Presence-absence data and statistical analyses

We used PIRATE v1.0 (91) on PGAP-annotated genomes to calculate the core and accessory genome (pangenome) by classifying shared paralogous and orthologous genes, followed by PCoA of this output using the “dplyr” v1.1.4, “vegan” v2.6.6.1 packages in RStudio to calculate Jaccard distances between all samples based on shared gene content. Pairwise distances were then transformed using the “cmdscale” function from the “stats” package and sketched using the “ggplot2” v3.4.4 package. Gene presence-absence plots were made using the “pheatmap” v1.0.12 package. Fisher’s exact test was performed on the number of COG genes on each of the four main plasmid types with at least *n* >5 genes across all four plasmids using the “fisher.test” function in the “stats” 4.3.2 package. We used a total number of genes in extrachromosomal elements in the *gsE* population as the background expectation for comparisons. Chi-squared tests were also run using the “stats” package using the “chisq.test” function. Pagel’s lambda was calculated using the “phylosig” function from the “phylotools” v2.1.1 package using the specifications method = lambda and test = TRUE.

### Gene-tree simulations

To assess patterns of plasmid inheritance, we created custom scripts to generate distributions of gene tree distances among genes randomly subsampled from within (or across) replicons in our population—with larger gene tree distances indicating more gene tree heterogeneity and thus increased horizontal (versus vertical) transmission. First, we generated a list of the core genes in each plasmid using PIRATE. For each replicon, we used custom R scripts to randomly subsample two sets of 100 genes (10 from the small core of the type IV/pSym) from each strain, align them, create two phylogenetic trees, and calculate the GRF distance between trees, then repeated this process over 1,000 random resamplings. We plotted these GRF distributions using the geom_density function in the ggplot2 package. Next, we repeated this process, comparing samples from each plasmid to the chromosome. Because the chromosome is necessarily vertically transmitted each generation, the distribution of chromosome-to-chromosome comparisons serves as a null expectation for the GRF distribution of a vertically inherited element in the presence of horizontal gene transfer, recombination, and gene tree uncertainty. The custom pipeline to run the simulations can be found at https://github.com/chaseschwarz/Phylogenetic-Congruence-Resampling/tree/main.

## Data Availability

Raw reads and assembled genomes are uploaded to the NCBI genome and SRA depositories SRA study # SRP069749, BioProject # PRJNA310138, and accession # JBRFPF000000000 - JBRFMW000000000

## SUPPLEMENTAL MATERIAL

Supplemental Data Sets mbio.02497-25-s0001.xlsb Data Sets S1 to S4.

Supplemental Material mbio.02497-25-s0002.docx Supplemental results, Tables S1 to S3, and Fig. S1 to S6.

ASM does not own the copyrights to Supplemental Material that may be linked to, or accessed through, an article. The authors have granted ASM a non-exclusive, world-wide license to publish the Supplemental Material files. Please contact the corresponding author directly for reuse.
