# Supplementary material for: Plasmid transmission dynamics and evolution of partner quality in a natural population of Rhizobium leguminosarum
Source: mBio. 2025 Nov 10;16(12):e02497-25. doi: 10.1128/mbio.02497-25 (PMC12691615; doi:10.1128/mbio.02497-25)

**SUPPLEMENTARY RESULTS**

*Strain phylogenetic categorization*: We reconstructed a phylogenetic tree from a core chromosome alignment (Fig. S1), which showed that 56 of our 62 strains formed a single clade identified as *R. leguminosarum* sensu stricto, or *genospecies* *E* (*gsE*) (35). Four of the 62 strains (061_N, 173_C, 209_N, 231_N) were identified as *gsB*/*Rhizobium johnstonii* (36), and were characterized within our population by low diversity in the chromosomes shared between these four strains, with only two SNPs separating 209_N from the others (Fig. S1). The remaining two strains, 717_N and 773_N, fell outside both *gsE* and *gsB* clades (Fig. S1). To further investigate the identities of strains Strain 717_N and 773_N, we first queried the NCBI non-redundant database using 16S rRNA and *dnaA* marker loci from each strain to identify the closest match with a complete genome in NCBI. For strain 717_N, this was *Rhizobium beringeri* TP13 (RefSeq: GCF_035971255.1; 100% and 96.45% for 16S and *dnaA*, respectively, and 93.6% genome-wide ANI). Strain 717_N was equally distinct from our *gsB* strain 061_N (94.2%) and the type strains for *gsB/R. johnstonii* (RefSeq: GCF_000009265.1, 94.0%) and its sister species *gsJ/R. acaciae* (RefSeq: GCF_025941625.1, 94.0%), suggesting 717_N should not be grouped with *gsE* or *gsB* for further analyses. For strain 773_N, the closest NCBI match using 16S and dnaA was *Rhizobium sp.* WYJ-E13 (RefSeq: GCF_018987265.1; 99.12% and 92.86% for 16S and *dnaA*, respectively, and 88.2% genome-wide ANI). Further comparisons to nearby taxa indicate that 773_N is also quite distinct genome-wide from *R. viscosum* (RefSeq: GCF_014873945.1; 88.7%) and *R. mesosinicum* (RefSeq: GCF_019511345.1; 87.8%), since all queries were below the threshold commonly used to group strains into species (91).

*Gene functional COG analysis:* Overall, COG analysis suggested that high-level distribution of functional gene content was similar across all plasmids (Fig. S6A). Their unique patterns of gene presence-absence variation (PAV) Together with the k-mer analysis and gene content, these phylogenies robustly support distinct groupings within type I and type IV plasmids (I-a, I-b, I-c, and IV-a, IV-b, IV-c, and IV-d; Fig. 1, 2 and Fig. S3A-D). Across all major plasmid types in the population, four COG categories were completely absent in the functional prediction (A, B, W, Y). The absence of these functions was not surprising as they relate to RNA processing (A), chromatin (B), extracellular (W), and nuclear (Y) structures, which are more commonly associated with eukaryotic or chromosomal processes. Fisher’s exact test was conducted comparing presence of COG genes for all categories across each plasmid. To calculate over/under enrichment of COG functions on plasmids we used the odds ratio of each COG function, using log(odds_ratio)>2.0 and log<-2 as a threshold for enrichment (Fig. S6B). We show that no function was under-represented, and that type IV plasmids are enriched in U and L genes (U_type_IV: log(Odds_ratio)=2.02, L_type_IV: log(Odds_ratio)=2.59). This is probably due to the presence of transposase, recombinase, integrase and conjugation-related genes found in type IV plasmids (Supplemental Material 1). Interestingly, enrichment of U and L functions reflect variable gene content as opposed to be representative of the type IV plasmid backbones, since core type IV plasmid genes do not belong to U or L. Next, we ran a chi-squared test for each gene (present in at least 5 strains) to test for overrepresentation of genes in N versus C strains. After correcting for multiple tests, the only gene associated with differing environmental conditions was a predicted alpha-beta folds hydrolase gene (N n=20, C n=9, p-value=0.041), part of a large gene family known to have catalytic bond-cleaving activity (92), which was present in type II plasmids.

**SUPPLEMENTARY FIGURES AND TABLES**

Table S1: List of conserved genes across all type IV plasmid (pSym) representatives, sorted by type IV region and alphabetical order. Type IV region is reflected on figure S6E.

Table S2: Size and strain of origin of accessory plasmids in the pangenome.


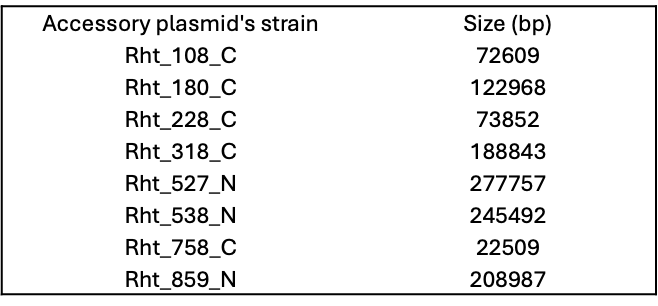


Table S3: Values for Pagel’s lambda, significant results are portrayed in bold, based on the type IV (pSym) phylogeny and greenhouse inoculation experiments phenotypic data as provided by Weese et al (2015).

| Replicon | Aboveground biomass | Chlorophyll content |
| --- | --- | --- |
| Chromosome | 7.33137e-05 | 0.73312 |
| Type I plasmid | 7.33137e-05 | 0.531772 |
| Type II plasmid | 0.363267 | 0.653511 |
| Type III plasmid | 7.33137e-05 | 0.436023 |
| Type IV plasmid | **0.61007** | **0.785386** |

Figure S1: Chromosomal phylogeny of clover-associated *Rhizobium* strains from the KBS LTER population alongside one representative each from five genospecies (*gsA-gsE*) of the *Rhizobium* species complex, as described in Cavassim et al. 2020. Tree was manually rooted on strain 773_N. Any nodes with < 85% bootstrap support were collapsed. A.

Figure S2: (A) Heatmap of pairwise Jaccard distances based on gene presence-absence between all plasmids from our study, highlighting similarity in gene content between plasmid types from *gsE* and *gsB*: I with V, II with VI, and III with VII/VIII. (B) Venn diagram of number of genes shared across plasmid types I and V, II and VI, and III, VII, and VIII. (C) local alignment of a representative of type III (strain 110_C) with representatives of types VII and VIII (both from 231_N).

A.


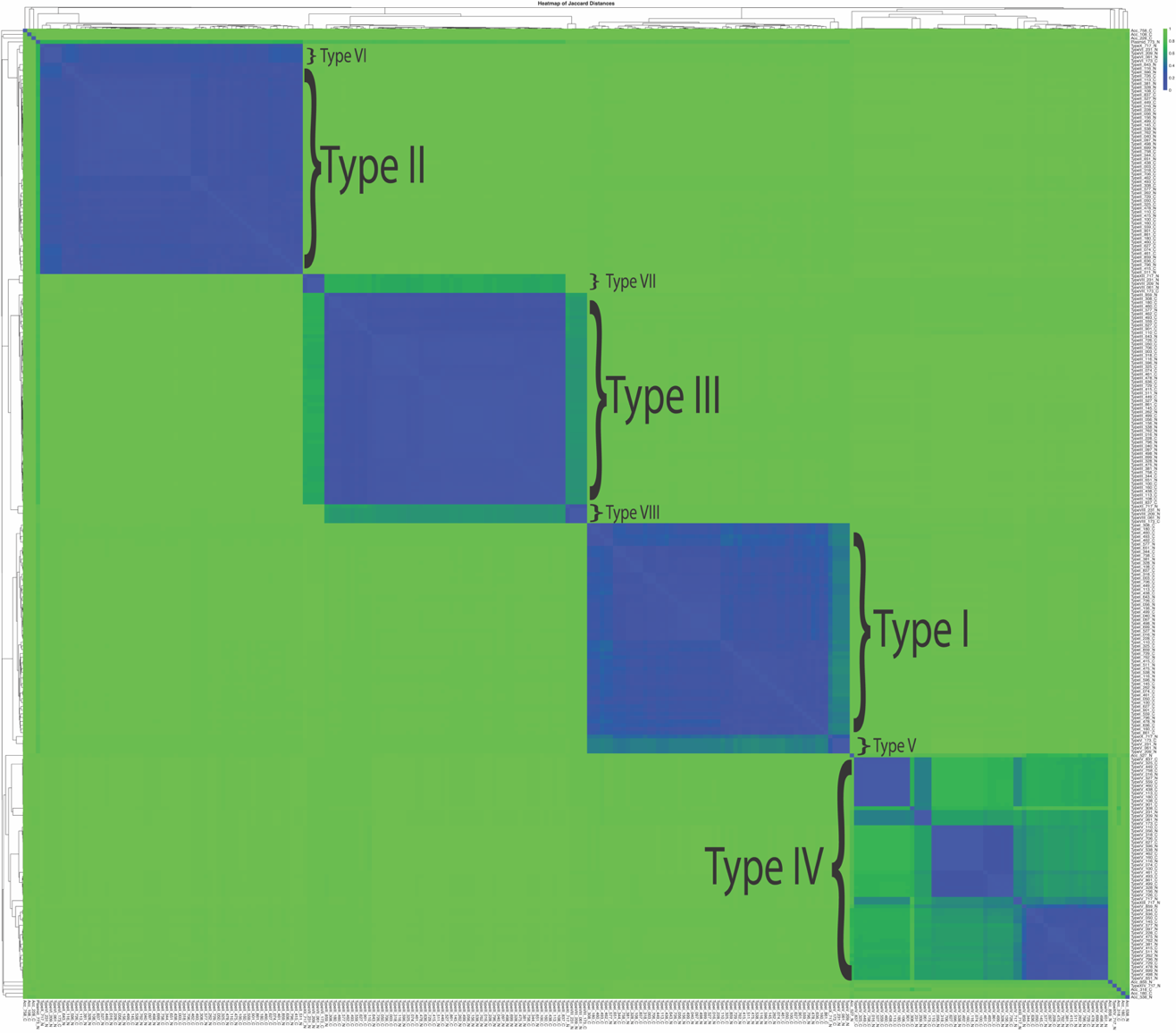


B.


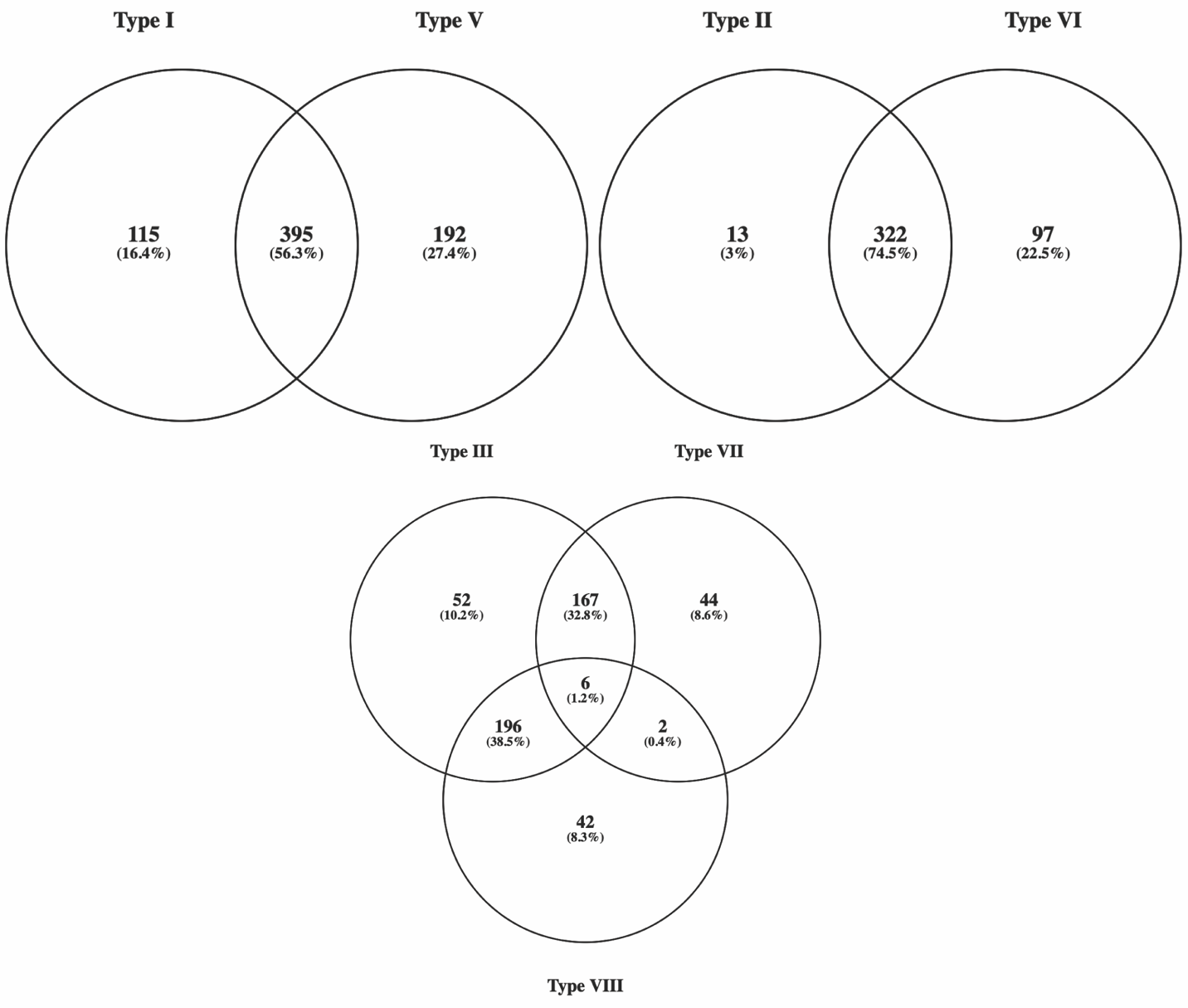


C.


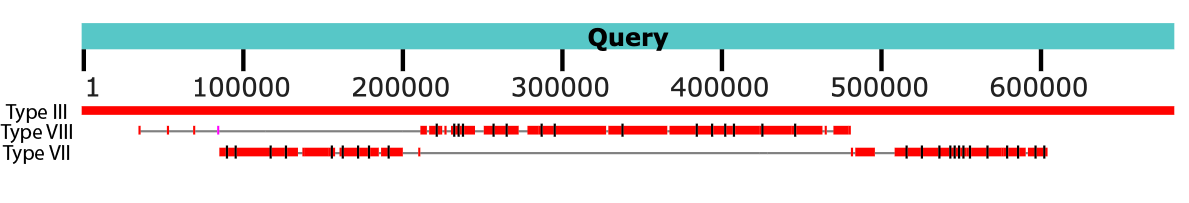


Figure S3: Tanglegrams and GRF distances of the inferred phylogenies between the chromosome and (A) type I plasmid (B) Type II plasmid (C) type III plasmid (D) type IV plasmid. We used cladograms instead of phylogenetic trees to generate tanglegrams because the original branch lengths were short, and our focus was on highlighting differences in topology, specifically how node splits vary between trees. To quantify these topological differences, we calculated the GRF distance, which compares node splits rather than genetic distances.

A.

B.

C.

D.

Figure S4: (A) Phylogenetic tree of the concatenated core genes of the type I plasmid, showing the relationships among the three type I plasmid sub-groups as in Figure 1. (B) Pangenome graph view of the Type I plasmid population, showing distinct insertions in larger subtypes I-b and I-c occur in the same genomic location. Aligned sequences are in black, and inverted regions are in red.

A.


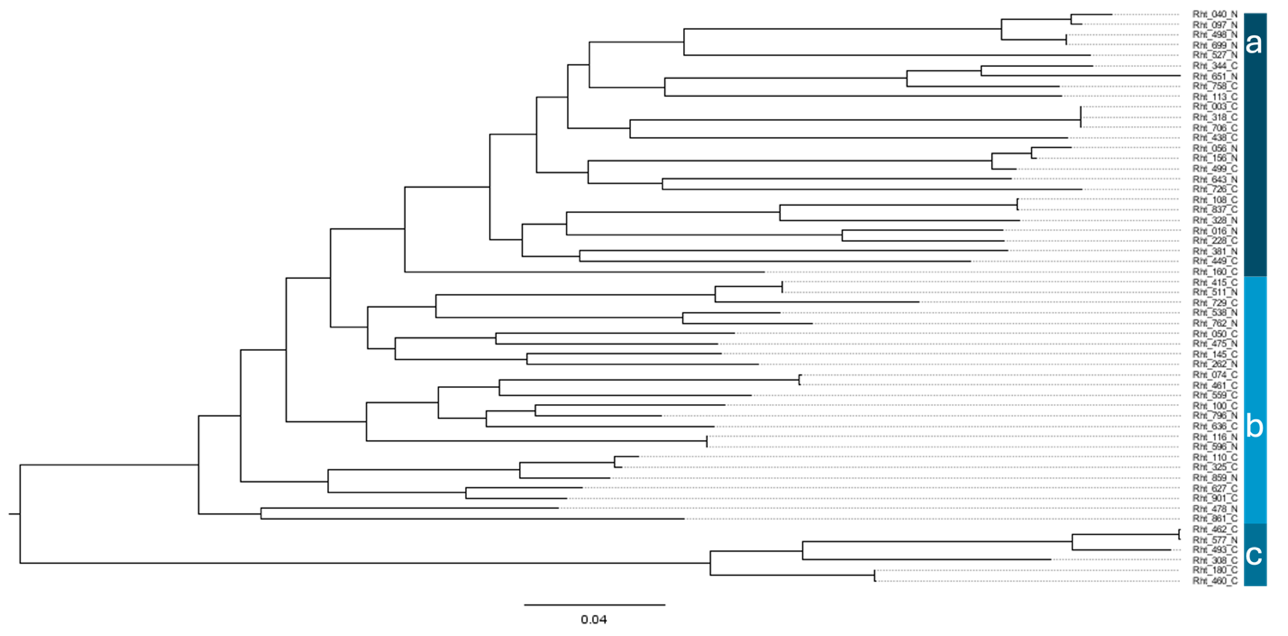


B.


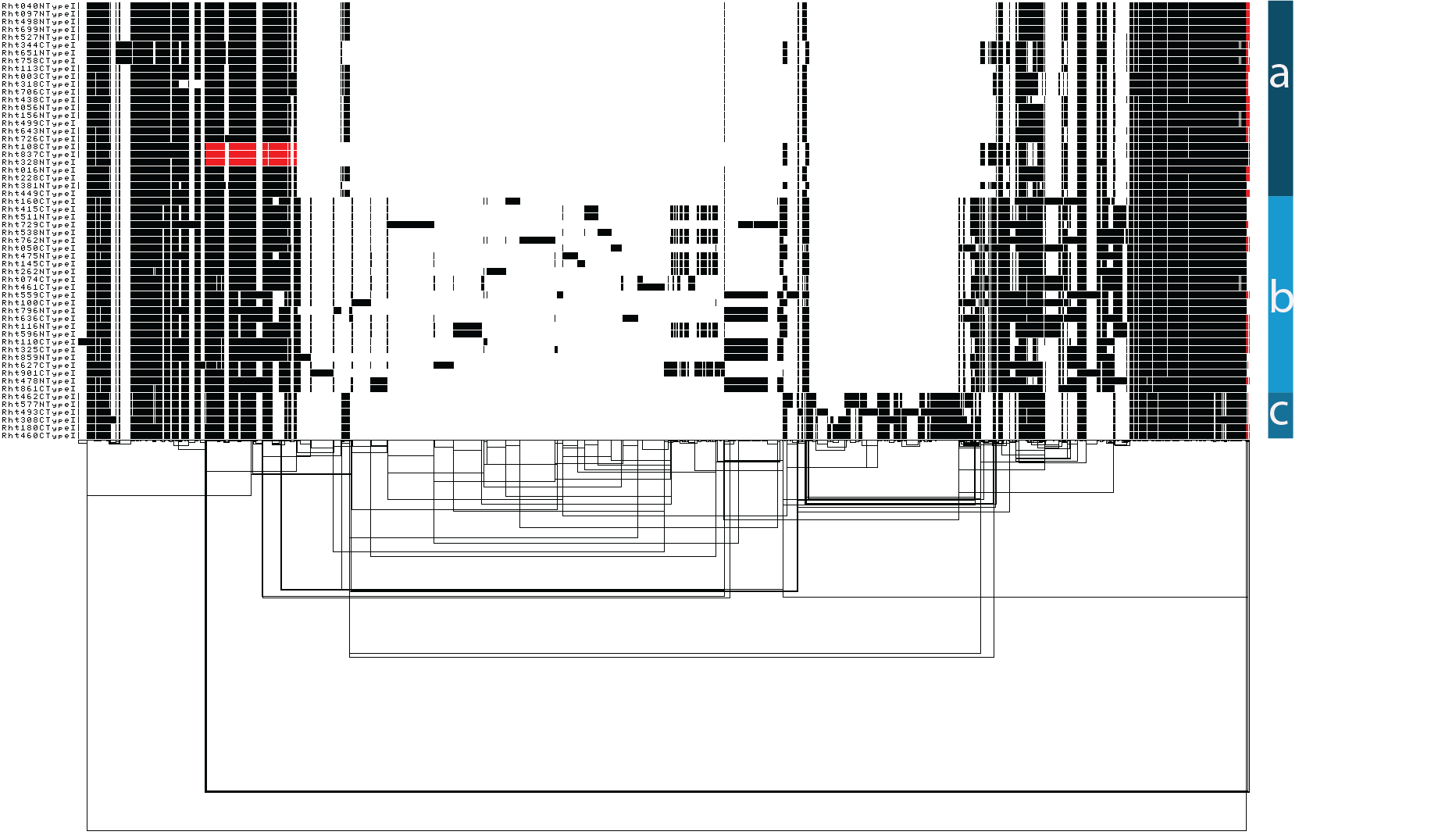


Figure S5: (A). Pairwise fixation index between aligned core components of subtypes of type IV plasmid a, b, and c (window size = 800 bp, step size = 200 bp), regions indicated in table S3 are represented by boxes underneath the sliding window analysis (blue=*(metal scavenging and ion transport region), gray=**(mobile element/transposon variable region), green=***(symbiosis region) (B) Principal Coordinate Analysis (PCoA) of orthologous gene clusters in the type IV plasmids in *Rhizobium.* (C) Inferred phylogenetic tree of type IV pSym showing orthologous genes that are present (red) or absent (blue) across strains. (D) Venn diagram showing the number of core genes in different combinations of type IV subtypes. (E-H) GRF distances within Type IV subtypes (smoothed distributions). (I) Inferred phylogenetic tree of type IV pSym showing Rh type presence in pSyms (as described by Cavassim et al. 2020).

A.


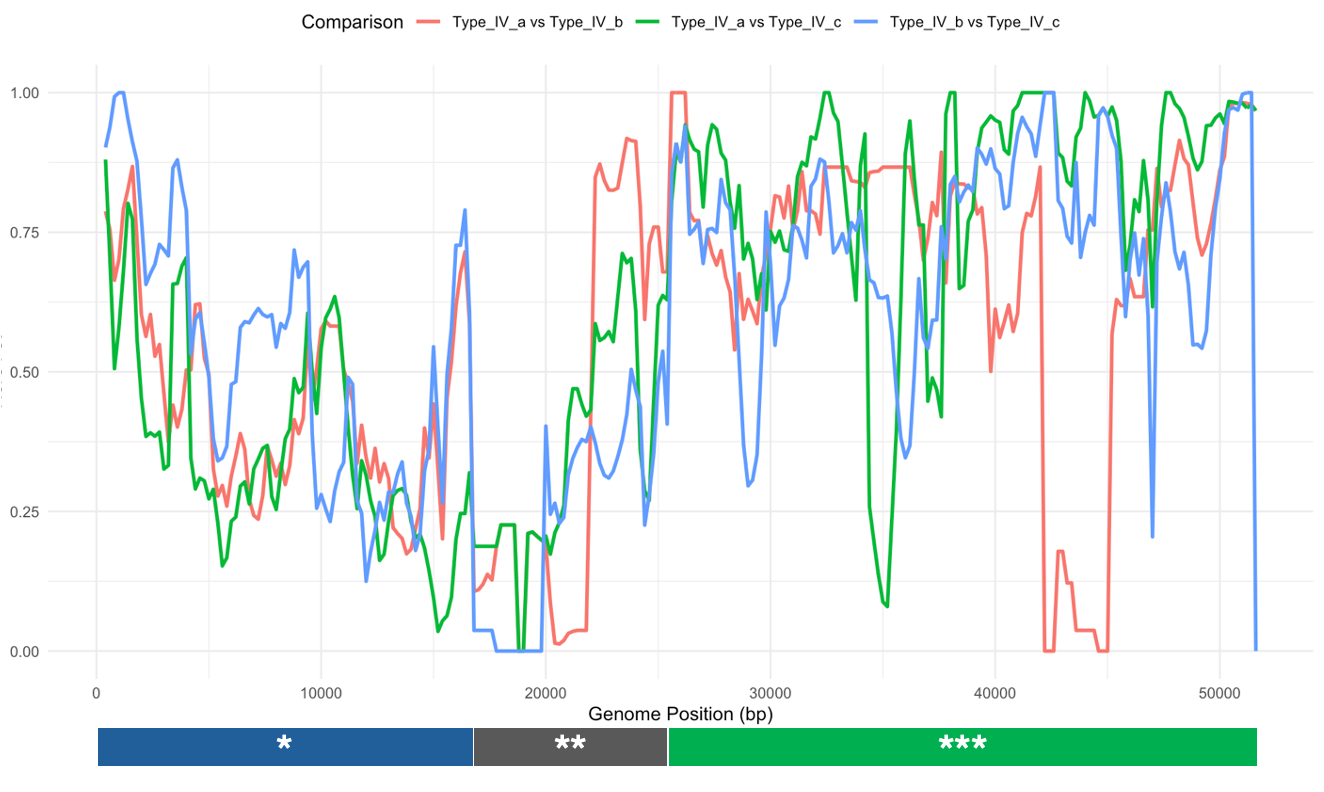


B.

C.


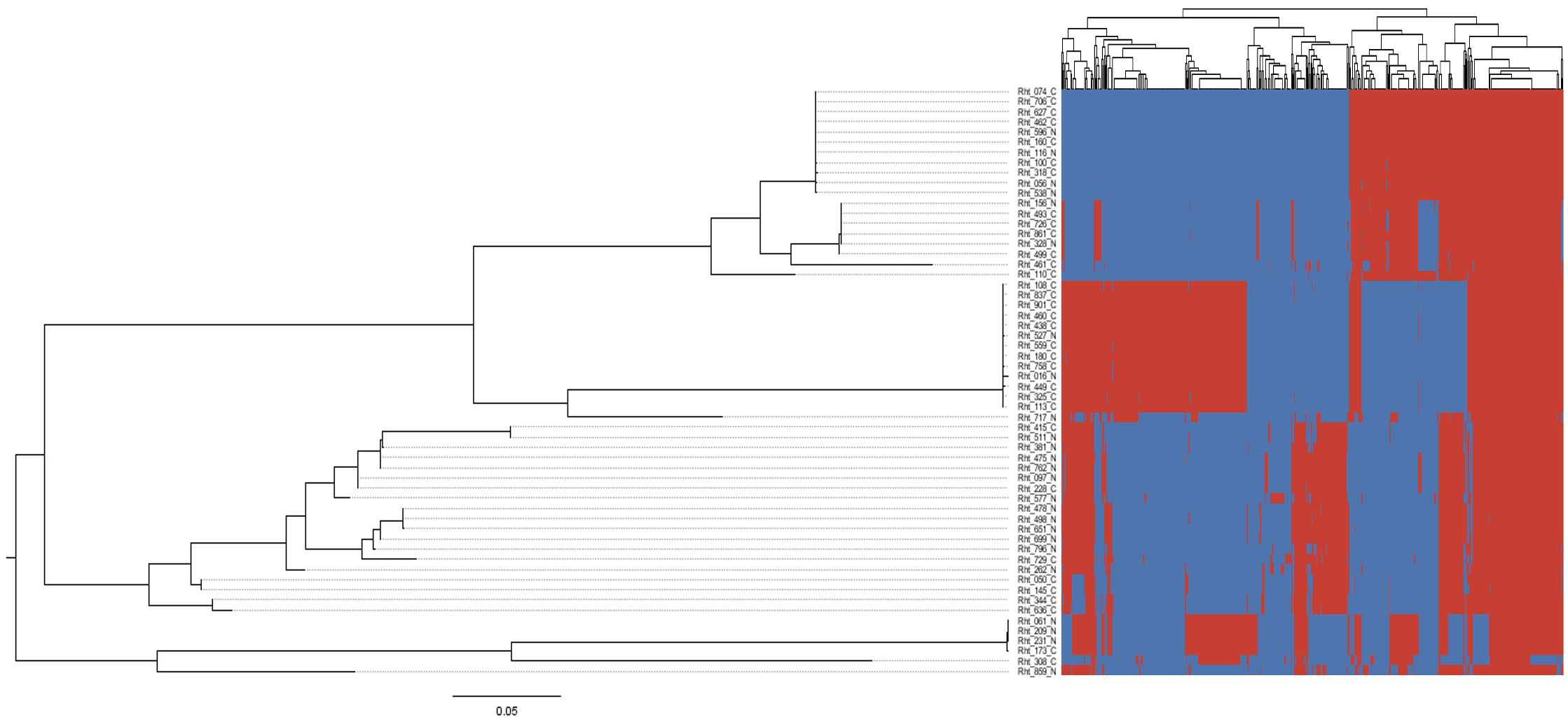

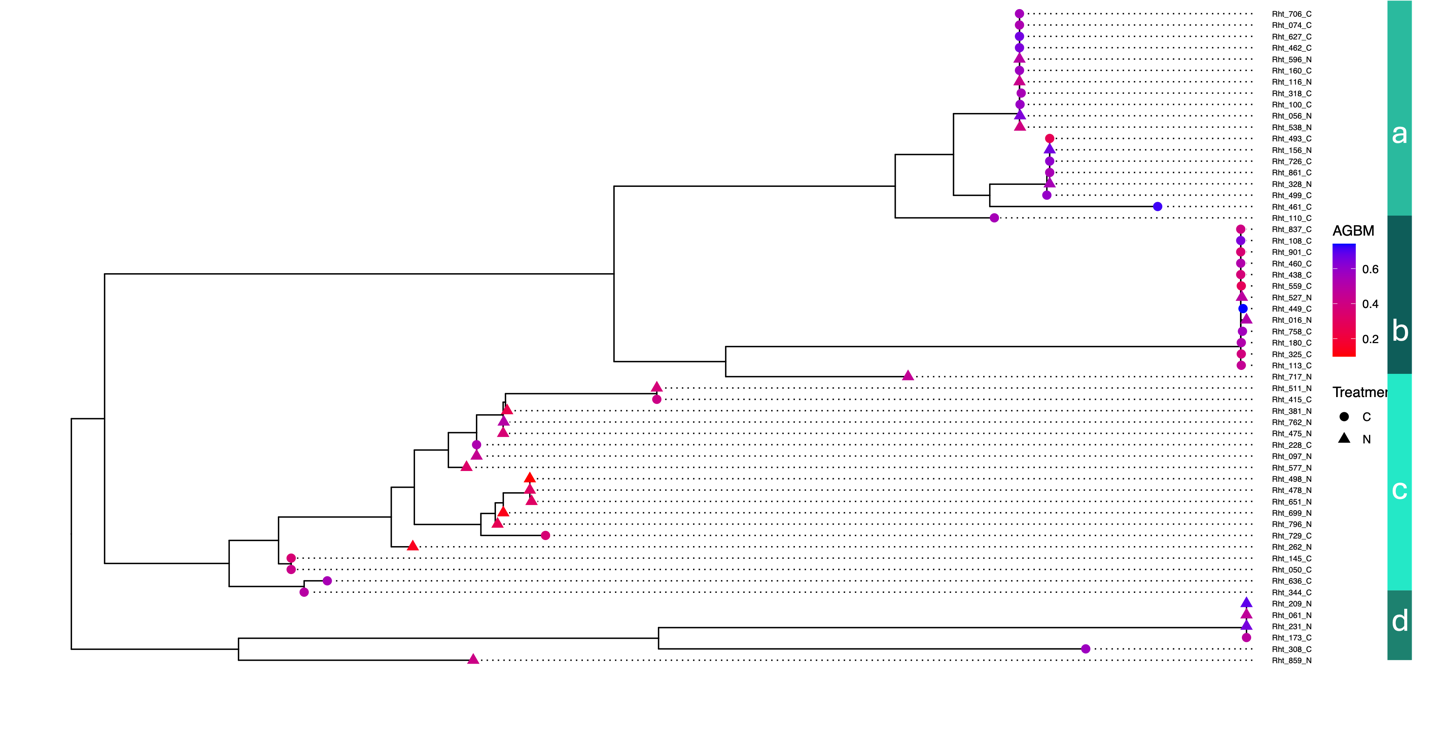


D.


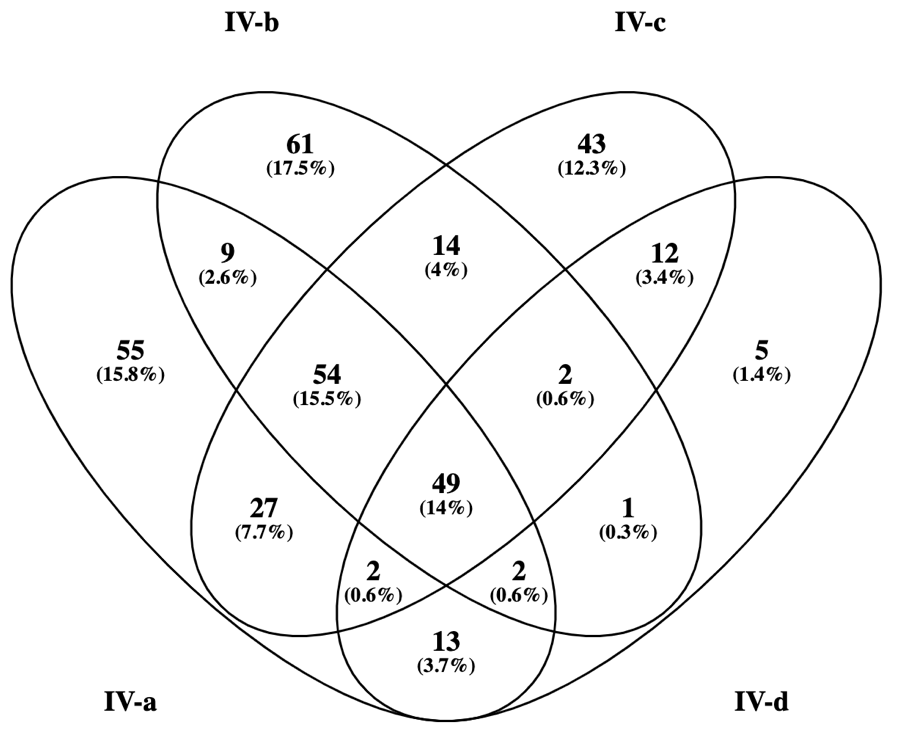


E-H.

**
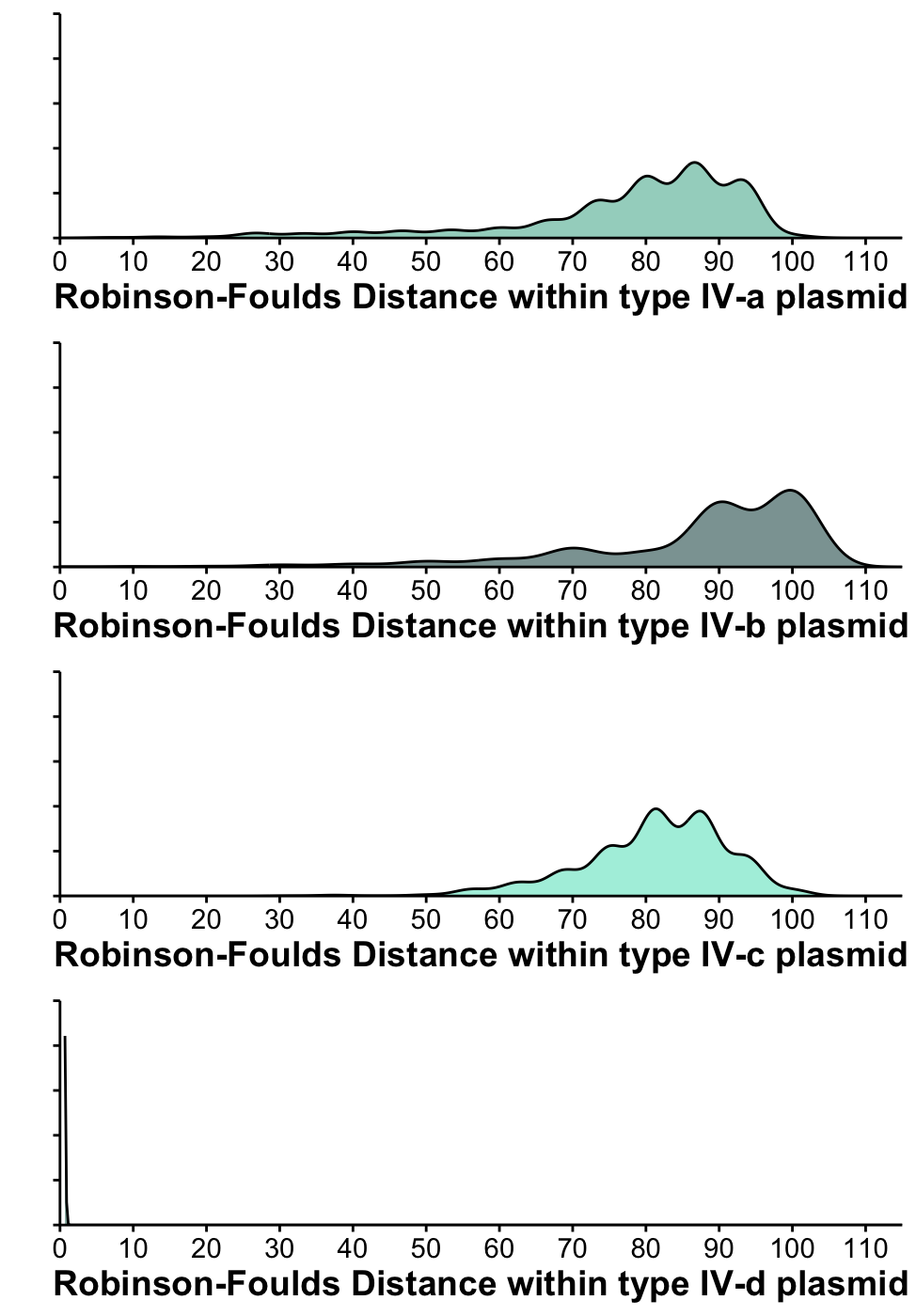
**

I.


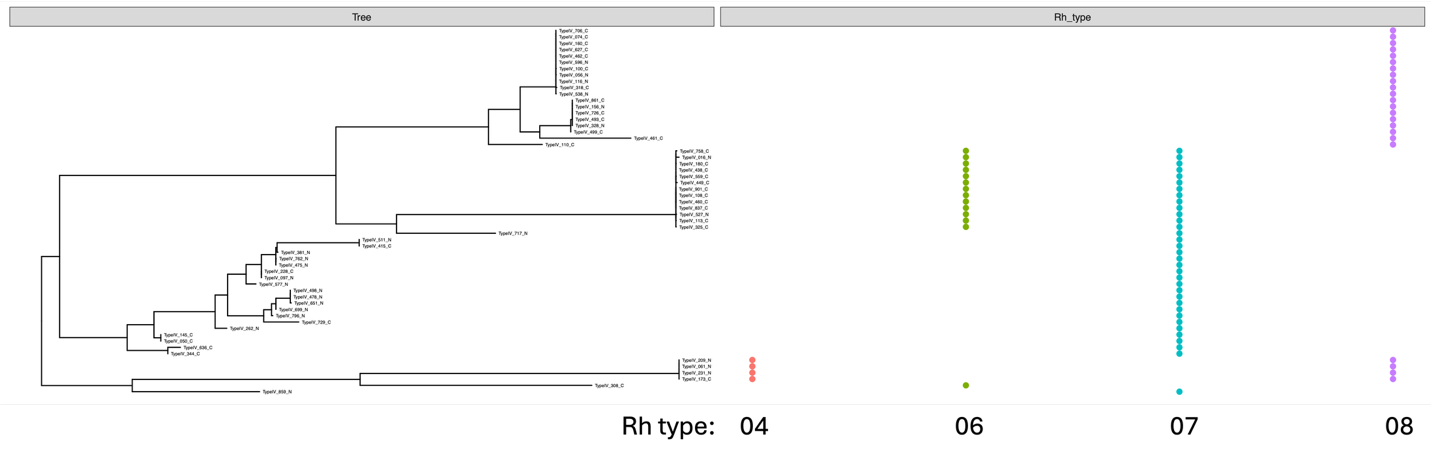


Figure S6: (A) Distribution of COG functions across the plasmids of clover-associated *Rhizobium* from a natural population: (S) Function unknown, (R) General function prediction only, (Q) Secondary metabolites biosynthesis, transport and catabolism, (P) Inorganic ion transport and metabolism, (I) Lipid transport and metabolism, (H) Coenzyme transport and metabolism, (G) Carbohydrate transport and metabolism, (F) Nucleotide transport and metabolism, (E) Amino acid transport and metabolism, (C) Energy production and conversion, (L) Replication, recombination and repair, (K) Transcription, (J) Translation, ribosomal structure and biogenesis, (B) Chromatin structure and dynamics, (A) RNA processing and modification, (Z) Cytoskeleton, (Y) Nuclear structure, (W) Extracellular structures, (U) Intracellular trafficking, secretion, and vesicular transport, (T) Signal transduction mechanisms, (O) Posttranslational modification, protein turnover, chaperones, (N) Cell motility. (B) Enrichment values of COG functions (log scale) in the 4 main plasmid types present in *gsE* strains. Enrichment was calculated as functions with a log odds ratio of over/under 2/-2, based on the proportion of functions associated in a specific plasmid compared to the sum total of extra-chromosomal COG functions.

A.
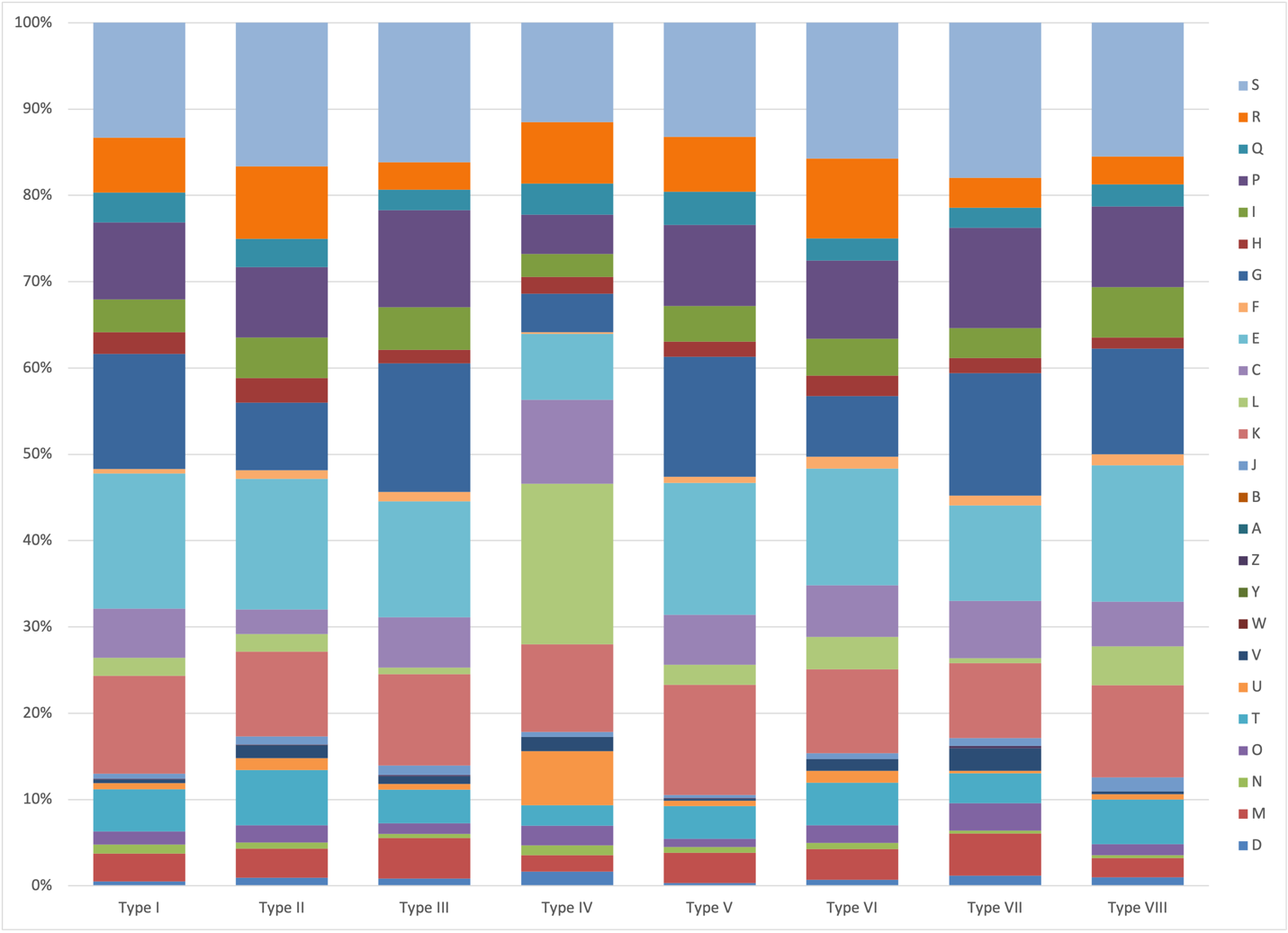


B.


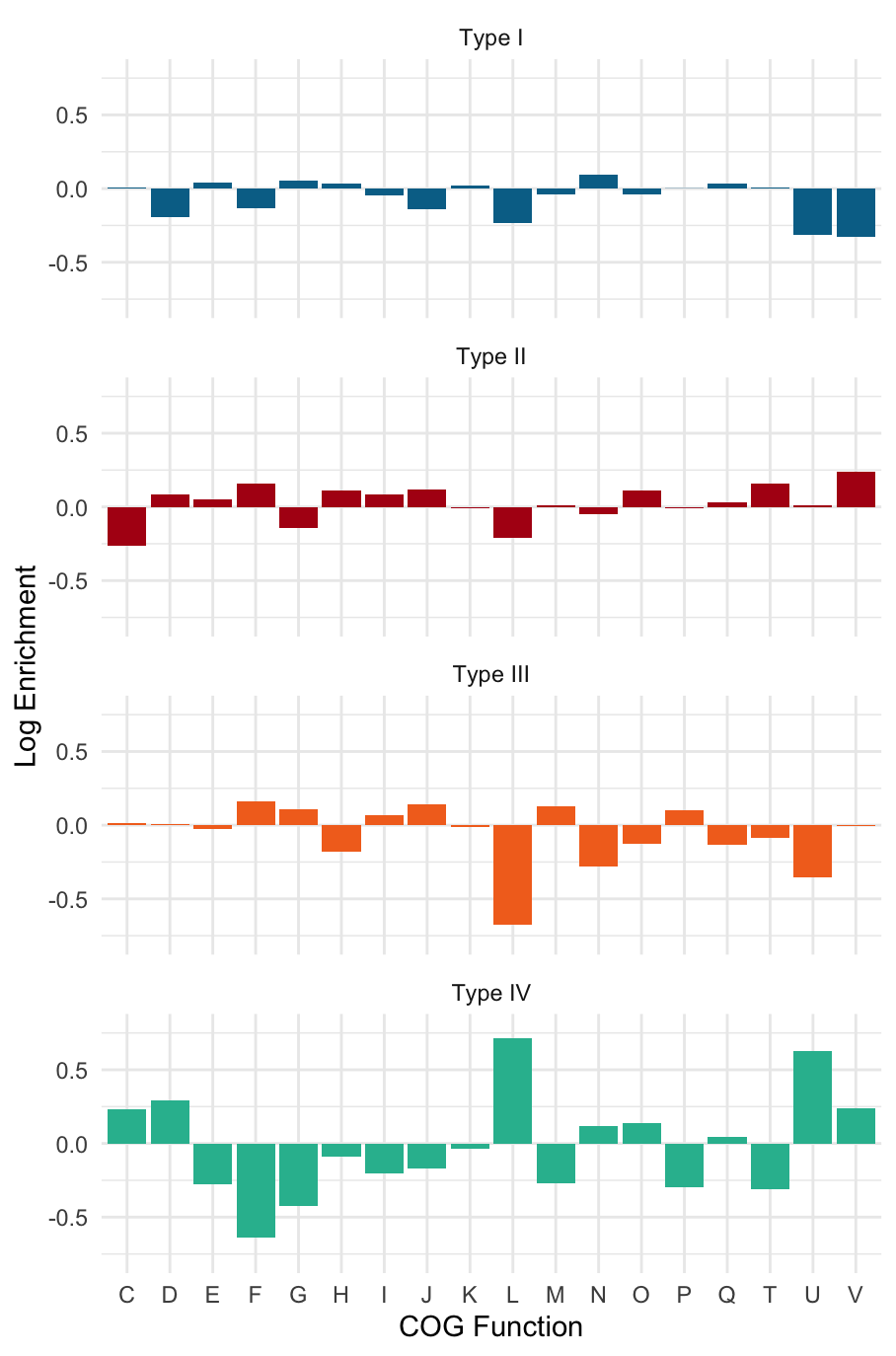

Supplement: Supplemental Material — Supplemental results, Tables S1 to S3, and Fig. S1 to S6. [file mbio.02497-25-s0002.docx]
